# Environmental methods for dengue vector control – A systematic review and meta-analysis

**DOI:** 10.1371/journal.pntd.0007420

**Published:** 2019-07-11

**Authors:** Claudia Buhler, Volker Winkler, Silvia Runge-Ranzinger, Ross Boyce, Olaf Horstick

**Affiliations:** 1 Institute for Medical Information, Biometry, and Epidemiology, Pettenkofer School of Public Health (PSPH), Ludwig-Maximilians University Munich, Munich, Bavaria, Germany; 2 Heidelberg Institute of Global Health, University of Heidelberg, Heidelberg, Baden-Wuerttemberg, Germany; 3 Division of Infectious Diseases, University of North Carolina at Chapel Hill, Chapel Hill, North Carolina, United States of America; Centers for Disease Control and Prevention, UNITED STATES

## Abstract

**Background:**

Vector control remains the primary method to prevent dengue infections. Environmental interventions represent sustainable and safe methods as there are limited risks of environmental contamination and toxicity. The objective of this study is to perform a systematic review and meta-analysis of the effectiveness of the following environmental methods for dengue vector control.

**Methodology/Principal findings:**

Following the PRISMA guidelines, a systematic literature search was conducted using the databases PubMed, EMBASE, LILACS, the Cochrane Library and Google Scholar. Quality assessment was done using the CONSORT 2010 checklist. For the meta-analysis the difference-in-differences (DID) and the difference-of-endlines (DOE) were calculated according to the Schmidt-Hunter method for the Breteau index (BI) and the pupae per person index (PPI). Nineteen studies were eligible for the systematic review, sixteen contributed data to the meta-analysis. The following methods were evaluated: (a) container covers with and without insecticides, (b) waste management and clean-up campaigns, and (c) elimination of breeding sites by rendering potential mosquito breeding sites unusable or by eliminating them. Study quality was highest for container covers with insecticides, followed by waste management without direct garbage collection and elimination of breeding places. Both, systematic review and meta-analysis, showed a weak effect of the interventions on larval populations, with no obvious differences between the results of each individual method. For the meta-analysis, both, container covers without insecticides (BI: DID -7.9, DOE -5) and waste management with direct garbage collection (BI: DID -8.83, DOE -6.2) achieved the strongest reductions for the BI, whereas for the PPI results were almost opposite, with container covers with insecticides (PPI: DID -0.83, DOE 0.09) and elimination of breeding places (PPI: DID -0.95, DOE -0.83) showing the strongest effects.

**Conclusions:**

Each of the investigated environmental methods showed some effectiveness in reducing larval and pupal densities of *Aedes sp*. mosquitoes. However, there is a need for more comparable high-quality studies at an adequate standard to strengthen this evidence.

## Author summary

Dengue is an emerging mosquito-borne viral disease, which is transmitted through the bite of *Aedes sp*. mosquitoes. The dengue virus causes 390 million infections and 96 million cases every year, and represents a major public health problem in the tropics and subtropics. Although a dengue vaccine was licensed in late 2015, it does not represent a general solution due to significant imperfections. Vector control remains still the primary method to prevent dengue infections. Environmental interventions to control dengue vectors represent sustainable and safe methods as there are limited risks of environmental contamination and toxicity. We provide a systematic review and meta-analysis of the effectiveness of the following environmental methods for dengue vector control: container covers with and without insecticides, waste management with and without direct garbage collection, elimination of breeding places. Both, systematic review and meta-analysis, showed a weak effect of the interventions on larval *Aedes sp*. populations, with no obvious differences between the results of each individual method. However, there is a need for more comparable high-quality studies at an adequate standard to strengthen this evidence.

## Introduction

Estimates suggest that there are about 390 million dengue virus infections and 96 million cases of symptomatic dengue per year [1]. Vector control remains the primary method to reduce dengue transmission, since initial optimism regarding the potential impact of a polyvalent vaccine has waned. [2].

Controlling *Aedes sp*., a cosmotropical mosquito with a preference for breeding in a wide range of artificial and natural containers with fresh and clean water, remains difficult [3–5]. Dengue control has become even more challenging because of urbanisation, population growth, increased international travel and lack of programme implementation for vector control in dengue-endemic countries [3, 6]. Environmental conditions in resource-limited settings such as weak infrastructure and poor sanitation also contribute to dengue transmission [3].

Vector control interventions can be distinguished into chemical, biological and environmental methods [3]. As for biological and chemical methods, these have been systematic reviewed with variable results [7]. Vector control strategies that aim to reduce breeding and proliferation of *Aedes sp*. mosquitoes by means of modifications to the environment are also critical. These methods may entail emptying or destroying water containers, cleaning of potential vector breeding sites, using container covers, applying waste management strategies, implementing community-based clean-up campaigns and the installation of piped water supply [3]. A primary advantage of these approaches is that they do not carry the risk of environmental contamination and toxicity and they are not vulnerable to the development of biological resistance, as is seen with many larvicides. Furthermore, the impact of environmental modifications is often long lasting and requires little further investment to sustain [8].

However, the overall role in reduction of dengue transmission of vector control remains unclear. This is partly due to issues about study design with many studies not having sufficient quality [9, 10], but also due to the fact that it is difficult to link vector control studies and transmission.

Two meta-analyses compared the effects of different dengue vector control methods, including most environmental methods, concluding there are too few studies at a sufficient standard [9, 10]. However, recent primary studies presented different results. Erlanger et al. concluded that environmental management was minimal effective on vector indices [10]. Bowman reported a reduced dengue risk for the combination of community-based environmental management interventions and the covering of water containers [9].

Therefore, the objective of this study is to systematically review and conduct a meta-analysis for the effectiveness of environmental dengue vector control methods. Specifically, we focus on: (a) container covers with and without insecticides, (b) waste management and clean-up campaigns, and (c) elimination of breeding sites by rendering potential mosquito breeding sites unusable or by eliminating them.

## Methods

This study follows the PRISMA guidelines for reporting systematic reviews and meta-analyses [11]. The literature search was performed until 30^th^ May 2017 in the following databases: EMBASE, Google Scholar, LILACS, PubMed and WHOLIS. Cochrane Library was searched for relevant reviews. The cited references of each included article were reviewed for potentially eligible studies. Data extraction was completed by one reviewer creating an evidence table (S1 Table). The eligibility criteria for the assessed literature were: (i) randomised controlled trials (RCTs) or cluster randomised controlled trials (cRCTs) with the following environmental vector control methods: (a) container covers without insecticides, (b) container covers with insecticides, (c) waste management with direct garbage collection, (d) waste management without direct garbage collection and (e) elimination of breeding places, (ii) outcome measures for pupal or larval indices, (iii) field studies conducted where dengue vectors naturally occur. Quality assessment was made using a tool modified from the CONSORT guidelines [12]. The Consort 2010 checklist was used as a framework for the identification of weaknesses and strengths in the reporting and structure of the trials. The complete quality assessment is available in the appendix.

For the systematic review, the descriptive analysis followed pre-defined categories of the data extraction (S1 Table). The analytical part followed content analysis [13]. Meta-analyses were done for the Breteau Index (BI) and the Pupae per Person Index (PPI) stratified by environmental method. Data from multi-country trials as well as data from multiple interventions within one trial were handled as separate studies. The difference-in-differences (DID) compares the differences before and after the intervention between the intervention and the control group. The difference-of-endlines (DOE) compares the endline measures between the intervention and the control group. Both effect measures were calculated for BI and PPI in each study using measures at baseline (b) and endline (e) separated by intervention (I) and control (C) group according to the following formulas. If multiple baseline values were available, their mean was used. To improve comparability, follow-up measurements that were closest to 12 months post-intervention were used. Many studies neither reported standard deviations nor confidence intervals, therefore, a pooled estimate weighted by sample size was calculated according to the Schmidt-Hunter method and visualised using forest plots [14]. Several authors were contacted to request missing data. All analyses were performed with the statistical software R version 3.4.1 (https://cran.r-project.org/bin/windows/base/old/3.4.1/).

BI_DID_ = (BI_ei_−BI_bi_)–(BI_ec_−BI_bc_)

PPI_DID_ = (PPI_ei_−PPI_bi_)–(PPI_ec_−PPI_bc_)

BI_DOE_ = BI_ei_−BI_ec_

PPI_DOE_ = PPI_ei_−PPI_ec_

No ethics approval was required because this secondary research is based on already published data.

## Results

### Study selection

**Fig 1:**
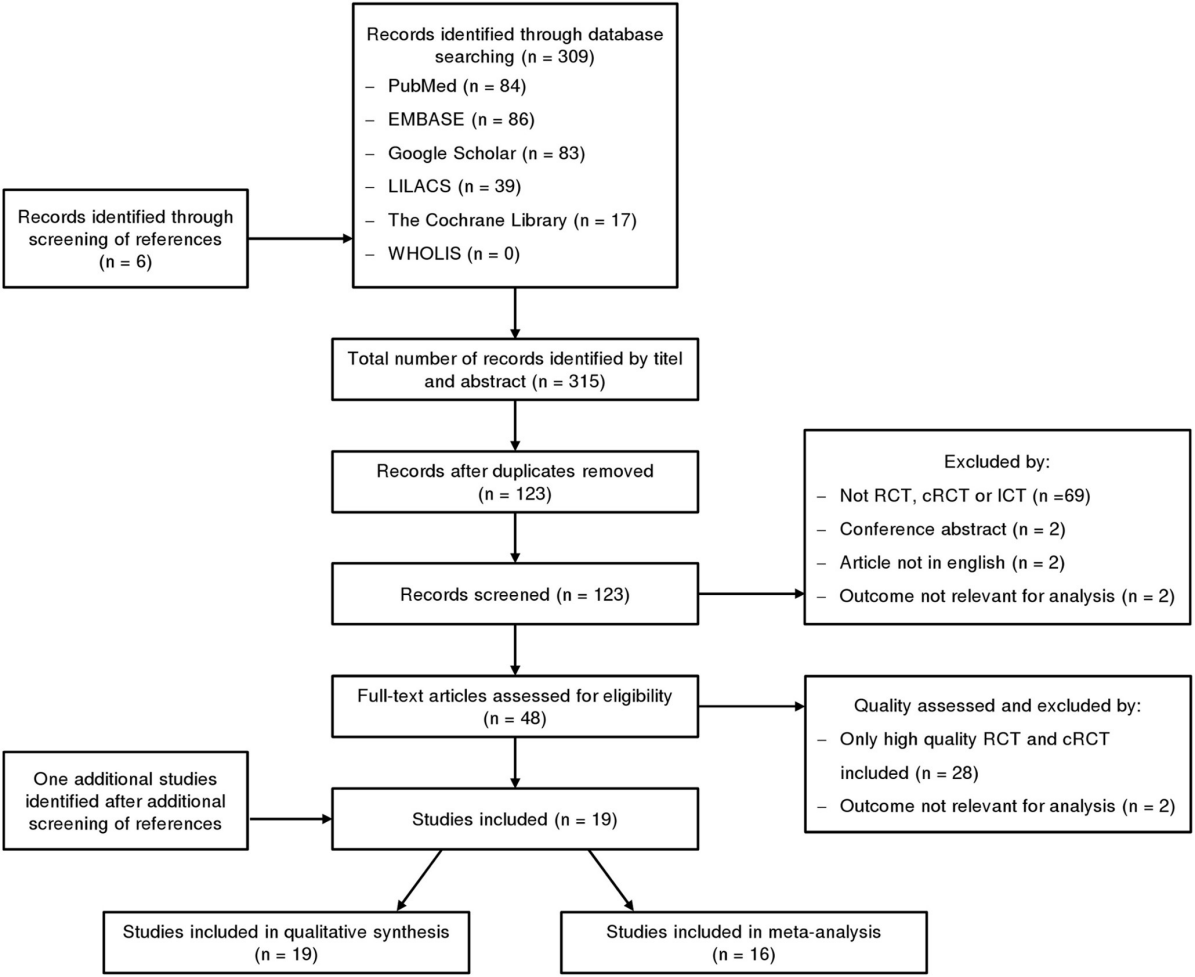
Flow diagramme. Diagramme describing the paper selection process based on the PRISMA flow diagramme.

A total of 309 articles and abstracts were identified through database searches and six additional articles were found by reviewing reference lists. After screening of duplicates, 123 records were initially screened by title and abstract, and 48 full-text articles were assessed further for eligibility. The most common reasons for exclusion from both systematic review and meta-analysis were: insufficient quality, only conference abstracts and study protocols, studies with the abstract published not in English and outcomes were not relevant for analysis (see Fig 1). One additional study was identified after a further screening of references. Nineteen studies were included in the systematic review [15–33], 16 (15–24, 26, 27, 29, 30, 32, 33) of which were included in the meta-analysis.

### General study characteristics

All included studies were published between 1993 and 2016. Geographically, most studies were conducted in South America (42%) (15, 17–20, 29b, 29d, 32b) and Central America (42%) (16, 23, 25, 29a, 30-32a, 33), with the remainder in South Asia (18%) (21, 22, 28) and Southeast Asia (21%) (24, 26, 27, 29c).

Except one RCT, all studies were designed as cRCTs. The sample size varied from two to 75 study clusters. Duration of the interventions ranged from six weeks to 30 months with seven studies (19, 21, 23, 25, 26, 31, 32) with a duration over one year, and the median duration was 6 months.

Twelve studies combined two or more vector control methods. Container covers without insecticides were implemented in eight studies (15, 17, 18, 22, 24, 27, 28, 31), more specifically all these studies used lids or nets on water storage containers. In six studies (15, 18, 22, 24, 27, 31), container covers were implemented in parallel with waste management campaigns.

Four studies (20, 25, 29d, 32a+b) installed container covers with insecticides. Kroeger et al. (31a) applied lambdacyhalothrin to curtains in Mexico, whereas the other study by Kroeger et al. in Venezuela (32b) used firstly deltamethrin and after five months additionally lambdacyhalothrin was applied. Two studies treated water with Temephos (25) and pyriproxyfen chips (32a).

Waste management and clean-up campaigns included community events, workshops, provision of information material, education on waste management, school programmes, community empowerment, capacity building, integrated vector management strategies, involvement of local stakeholders, provision of waste collection bags, garbage collection and rubbish disposal. These interventions were performed by professionals and community members. The waste management category was divided into two sub categories, with direct garbage collection (eight studies) and no garbage collection (five studies). Direct garbage collection means that cleaning activities were included in the waste management programme, whereas studies with no direct garbage collection only provided information, education and training material.

Elimination of breeding places was used in five studies (17, 24, 26, 29b, 30). Applied interventions included emptying buckets, periodic inspection of houses and elimination or protection of disposable items and discarded or unused water holding containers.

Seven studies also implemented non-environmental methods as insecticide-treated curtains for windows and doors (15, 20, 25), copepods (24), *Bacillus thuringiensis israelensis* (24, 27), fish (16), dragonfly nymphs (27), mosquito traps (24), portable vacuum aspirators (24), cotton net sweepers (27) and container treatment with insecticides (15, 25, 27, 29b).

The control groups of four studies were untreated and no dengue vector control interventions were conducted. All other studies used routine programmes as control (15, 28, 32, 33). Most of them were already existing governmental dengue vector control programmes as entomological surveillance, health education, source reduction through periodic house inspections and application of larvicides and adulticides.

### Outcome measures

A total of 17 studies reported BI (15–27, 29, 30, 32, 33), 15 studies reported House Index (HI) (15–22, 24–26, 30–33), 13 studies reported Container Index (CI) (15–18, 20–22, 24–27, 31, 32), 15 studies reported PPI (15–22, 24–27, 29, 30, 32) and five studies reported the number of positive containers (17, 20, 24, 28, 31). Only the study by Overgaard et al. (15) reported the number of mosquitoes per hour inside schools collected with a Prokopack aspirator. Three studies reported measures for human transmission as the number of pupil absence periods at school (15), cluster specific rates of dengue virus infection in paired saliva samples from children and the number of reported dengue cases (16) and IgM serology (32).

Studies provided no or only limited information regarding larval sampling methods. Only four studies (16, 19, 26, 28) reported sufficient surveillance data to allow reproducibility.

All studies conducted one or two baseline surveys and reported at least one entomological outcome measure. The discrepancy in outcome measures between intervention and control groups varied across the studies but about one third had a difference of at least 40% (BI: 15, 20, 26, 29b, 32a; PPI: 15, 17, 18, 20, 26, 29a+b, 32a+b).

Eight studies conducted the entomological baseline survey and follow-up surveys during distinct dengue transmission seasons (17, 18, 23, 24, 27, 29a, 32b, 33). Three studies (17, 18, 24) conducted the baseline survey during low transmission and the endline survey during high transmission season and the others vice versa.

### Results of the systematic review

#### Container covers without insecticides

Within the category container covers without insecticides, only the study by Overgaard et al. [15] was of high quality (see S3 Appendix. Quality Assessment). The authors reported greater reductions in intervention clusters (CI: 2.2, PPI: 0.04 at endline) compared to the control areas (CI: 7.1, PPI: 0,36 at endline). However, they reported an increase for PPI in both intervention and control clusters, with higher levels of PPI in control clusters. The studies by Basso et al. [17] and Arunachalam et al. [22] were of medium quality (S3 Appendix Checklist). Arunachalam et al. reported consistent positive results, whereas Basso et al. demonstrated an increase in vector densities from baseline to endline, with higher indices in control clusters. BI increased in intervention clusters from 3.4 to 12.02 and in control clusters from 2.64 to 13.77. Five studies [18, 24, 27, 28, 31] were considered of low quality. The results of these studies are summarized in S1 Table.

#### Container covers with insecticides

The studies by Quintero et al. [20] and Kroeger et al. [32] were of high quality. Quintero et al. [20] reported a significant reduction in intervention clusters and an increase in control clusters for PPI after the second intervention. Larval densities were only slightly reduced compared to the control. The study by Kroeger et al. [32] showed a significant decrease in entomological indices with no significant differences at endline between both groups, control (BI: from 113/34 to 12/17) and intervention (from 60/38 to 7/11).

Studies of medium quality were conducted by Rizzo et al. [25] and Tun-Lin et al. [29] in Venezuela. Both reported variable results. The study by Rizzo et al. [25] showed greater reductions in almost all entomological indices with the exception of CI where no significant differences were measured. Tun-Lin et al. [29] reported almost no differences for PPI and a similar increase for BI in both, intervention and control group (S1 Table).

#### Waste management with direct garbage collection

The studies that examined waste management with direct garbage collection are almost the same studies that also considered container covers without insecticides. Except of the study by Kusumawathie et al. [28], the same high- and low-quality studies are available.

The additional studies by Abeyewickreme et al. [21] and Castro et al. [23] were of medium quality (S3 Appendix), reporting lower entomological indices for intervention clusters at endline compared to control clusters. The study by Abeyewickreme et al. [21] measured discrepant results as HI and CI did not show differences between the groups (S1 Table). Castro et al. [23] reported an increase in BI from baseline to endline with a BI 53% lower in intervention clusters.

#### Waste management without direct garbage collection

Vanlerberghe et al. [30] conducted a high-quality study with large reductions in intervention clusters compared to control clusters (50% lower for HI and BI, 73% lower for PPI). The study by Andersson et al. [16] is also of high-quality although the baseline data are missing. They also reported greater reductions in all entomological indices.

The results of both medium-quality studies are highly variable. Mitchell-Foster et al. [19] reported a protective effect for the interventions as there were bigger reductions in all entomological indices. The study by Tana et al. [26] also demonstrated a protective effect of dengue vector control interventions despite the results were not completely consistent.

The study by Leontsini et al. [33] was of low quality although reporting promising results. Larval indices decreased in intervention clusters and increased in control clusters. Tun-Lin et al. in Mexico [29] and in the Philippines [29] could not be considered for a classification into one of the two waste management categories because of missing data about the applied interventions. Both were of medium quality with completely different results. In Mexico, the BI increased more in control cluster than in intervention clusters and the PPI decreased in intervention areas but increased in control areas. In the Philippines, there were very large reductions in both indices similar for both groups, intervention and control (S1 Table).

#### Elimination of breeding places

Except the study by Tun-Lin et al. in Peru [29], all studies implementing interventions for the elimination of breeding places (as elimination of water containers, emptying buckets or source reduction) also used further methods belonging to at least one of the other environmental intervention categories (S1 Table). Only the study by Vanlerberghe et al. [30] was of high quality. Three studies [17, 25, 29] were of medium quality and one study [24] was of low quality. Overall, two studies reported a protective effect of the intervention [25, 30] whereas three studies did not show homogenous results [17, 24, 29]. Tun-Lin et al. in Peru [29] did not show homogenous results.

Overall, the highest quality was assessed for container covers with insecticides, followed by waste management without direct garbage collection and elimination of breeding places. The lowest study quality was seen for container covers without insecticides and waste management with direct garbage collection (S3 Appendix).

### Meta-analysis of BI and PPI outcomes

**Fig 2:**
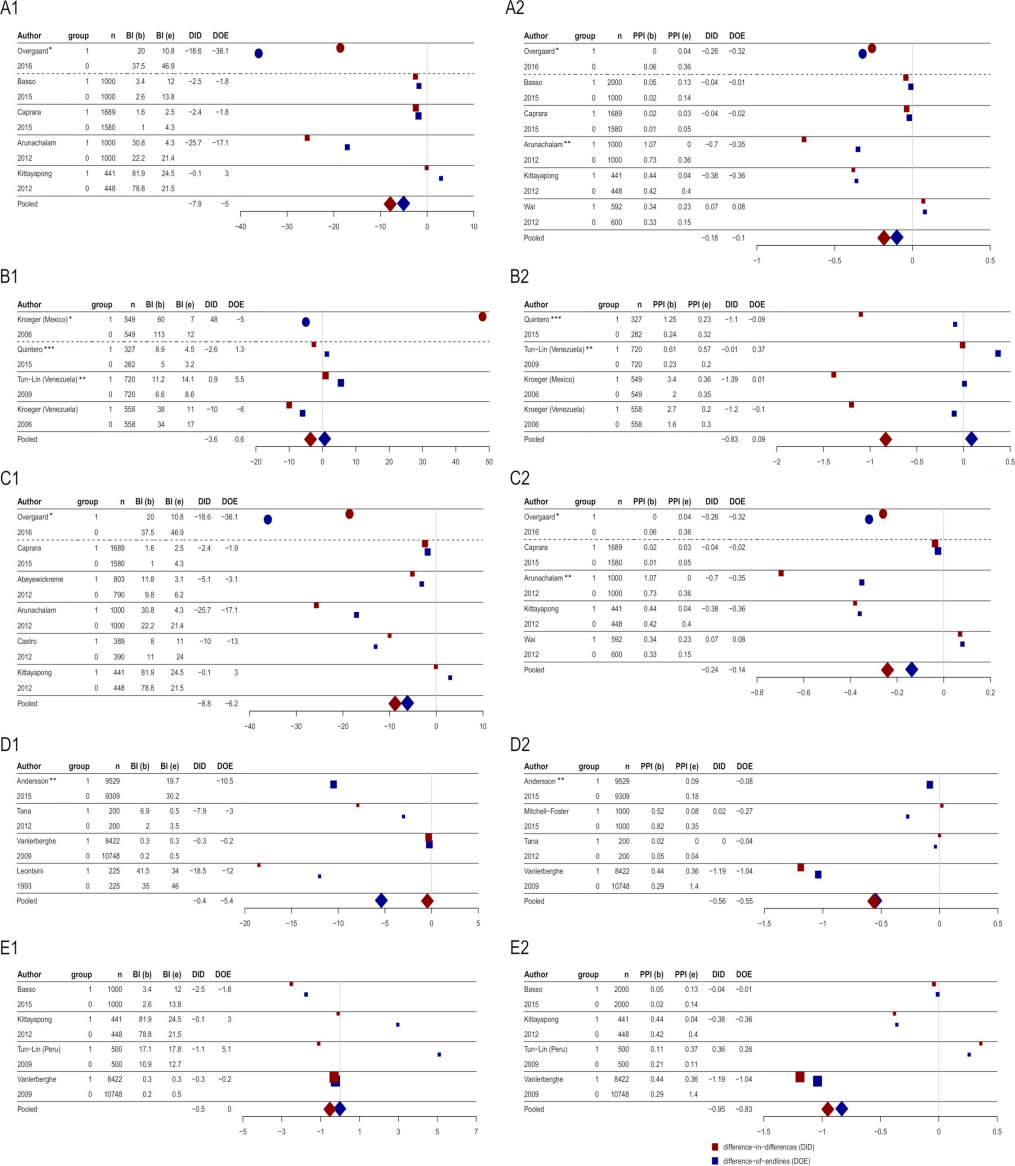
**A1: Forest Plot of container covers without insecticides for the outcome Breteau Index. A2: Forest Plot of container covers without insecticides for the outcome Pupae per Person Index. B1: Forest Plot of container covers with insecticides for the outcome Breteau Index. B2: Forest Plot of container covers with insecticides for the outcome Pupae per Person Index. Fig 2C1: Forest Plot of waste management with direct garbage collection for the outcome Breteau Index. C2: Forest Plot of waste management with direct garbage collection for the outcome Pupae per Person Index. D1: Forest Plot of waste management without direct garbage collection for the outcome Breteau Index. D2: Forest Plot of waste management without direct garbage collection for the outcome Pupae per Person Index. E1: Forest Plot of elimination of breeding places for the outcome Breteau Index. E2: Forest Plot of elimination of breeding places for the outcome Pupae per Person Index.** For each category, the difference-in-differences and the difference-of-endlines are presented. *Result was not included in summary measurement. **Result differs from publication. ***Method was implemented in a subsample as a second additional intervention.

The results within the individual categories, the studies showed a high discrepancy (Fig 2). However, most of them resulted in lower entomological indices compared to the control groups. Regarding the DID, only the studies by Tun-Lin et al. in Venezuela (29b) (+0.9) and Kroeger et al. in Mexico (32a) (+48.0) resulted in a negative effect for BI, whereas Mitchell-Foster et al. (19) (+0.02), Wai et al. (27) (+0.07) and Tun-Lin et al. in Peru (29a) (+0.36) resulted in a negative effect for PPI. Compared to the DOE, the individual studies showed strong discrepancies to the DID. The study by Kroeger et al. in Mexico (32a), measuring extremely different baseline values, showed an almost twice as much BI reduction in the control group.

The pooled results from all environmental intervention categories targeting dengue vectors measured as BI show that container covers without insecticides (-7.9) and waste management with direct garbage collection (-8.83) achieved the strongest reductions of the pooled DID for BI. Covers with insecticides also showed a strong reduction for BI from baseline to endline (-3.6) whereas waste management without direct garbage collection (-0.45) and elimination of breeding places (-0.53) had smaller impact.

Compared to BI, the differences in the PPI showed almost opposite results. Container covers with insecticides (-0.83) and elimination of breeding places (-0.95) showed the most protective effect. Covers without insecticides (-0.18) and waste management with direct garbage collection (-0.24) resulted in the lowest effect compared to the other interventions.

The results of the DOE tended to demonstrate a smaller impact on vector indices compared to the DID. In the case of container covers with insecticides, they showed completely discrepant results. Container covers with insecticides showed lower entomological indices at endline resulting in a difference-in-differences of -3.6 for BI and -0.83 for PPI. However, the endline indices were higher in intervention clusters compared to control cluster expressed by a DOE of +0.6 for BI and +0.09 for PPI.

## Discussion

This study evaluating the effectiveness of different environmental dengue vector control methods suggest that environmental interventions can be effective in controlling the immature stages of *Aedes sp*. mosquitoes. Most studies showed an effect of the interventions on larval populations, with no obvious differences between the results of each method. However, as in all vector control interventions regarding dengue the link between the reductions of vector populations and risk of transmission is not clear.

As for the specific methods, studies analysing container covers with and without insecticides showed mixed results. The variability of results has also been shown by Erlanger et al. [10] and Bowman et al. [9]. Some demonstrated the effectiveness of the interventions whereas other studies could not show a clear impact or even no effect on vector indices. The findings are unexpected since in theory, container covers with insecticides should result in an additional control effect, with a mechanical barrier for oviposition and adult emergence, but also with an insecticidal effect. Possible reasons why our results did not show the same, are the followings: (i) studies used particularly good sealing covers without insecticides, (ii) the insecticides used for the container covers were not applied correctly, and (iii) the *Aedes sp*. vectors were already resistant against the applied insecticides [34, 35].

Studies analysing waste management with direct garbage collection were almost the same studies using container covers without insecticides and thus, reported similar results. Bowman et al. [9] showed a reduced risk of dengue incidence for combining water container covers with community-based environmental management interventions (Container Index: Odds Ratio 0.22, 95% CI 0.15, 0.32). Since most studies combined both categories, we report similar results (BI: DID -8.8; DOE: -6.29). However, this intervention is often applied in practice anyway, with possible synergies for the control of other diseases.

The results of studies using waste management without direct garbage collection were more promising (BI: DOE -5.4). The systematic reviews by Erlanger et al. [10] and Bowman et al. [9] concluded with similar results. Erlanger et al. [10] also showed the effectiveness of education and waste management in the community on reducing vector densities. Bowman et al. [9] also concluded that community-based combination interventions including clean-up campaigns and mobilisation significantly impacted larval indices as shown for community-based combination interventions in Cuba (BI: rate ratio 0.48, 95% CI 0.26, 0.89). However, there is a discussion following Mitchell-Foster et al. [19] that evaluation of pedagogical approaches can change household level behaviours, resulting in a reduction in suitable habitat for juvenile mosquitoes [19]. Furthermore, we analysed if studies stratified by regular garbage collection as Mitchell-Foster [19] did it, which leads to different interpretation of the results. However, no such information was available in the other studies.

Except of Tun-Lin et al. in Peru (28b), all studies (16, 23, 24, 29) using elimination of breeding places implemented further environmental methods indicating that eliminating breeding sites alone is not considered effective which was also concluded by Bowman et al. [9] and Erlanger et al. [10]. The results across the studies were not homogenous as already shown by the other environmental methods.

The results of the systematic review show that each individual method was analysed by studies with post negative and positive outcomes. However, in general, lower entomological indices were reported for intervention clusters compared to control clusters.

The results of the meta-analysis also point to a positive effect of environmental methods towards reduction of larval and pupal density. Although the study by Erlanger et al. [10] reported only a minimal effect of environmental management on vector indices (BI: relative effectiveness 0.71%, 95% CI 0.55, 0.90), the results of Bowman et al. [9] and Erlanger et al. [10] point to the same finding as our analysis did: environmental control interventions can reduce dengue vector indices. Additionally, they did not compare as many intervention categories as we did and further, we included more studies implementing the respective methods. Overall, we could show how diverse the field of relevant studies is and how discrepant the results are. Environmental vector control strategies could be effective, however missing study comparability limits our results.

Despite including studies with high quality design, our study still has limitations compared to Bowman et al. [9] and Erlanger et al. [10].These limitations include a possible publication bias as systematic reviews and meta-analysis are only able to work with already published data and if an author did not represent all results, distortions are possible. This problem was difficult to avoid as only a few authors answered on our requests. However, the very broad search, containing grey literature as well, attempted to limit this bias. The meta-analysis was limited to the vector indices BI and PPI. Other indices were also available but not so often across the studies. Additionally, BI and PPI representing larval and pupal appearances are adequate to give a good overview. Unexpectedly, both indices showed almost opposite results. Maybe after the elimination of containers, the remaining ones are more productive and therefore influence the PPI.

Regarding the eligibility criteria described in the methods section, only a limited amount of studies was included in this work. Most studies concerning dengue vector control did not use a high-quality design or focused on non-environmental methods like chemical and biological interventions. Study quality was a major issue, with most studies assessed as low or medium quality. Only five studies (14, 15, 19, 29, 31) were of high quality. Except of Kroeger et al. (31), these high-quality studies showed positive results with much lower entomological indices for intervention clusters at endline. Studies with lower quality assessments had inconsistent results, with more studies reporting negative results on larval densities.

Different follow-up periods, sample sizes and settings concerning climate, altitude and population density might have distorted the comparability of data reported in the included studies. Similar effects can be observed in other published meta-analysis on vector control studies. Additionally, eight studies (16, 17, 22, 23, 26, 28a, 31b, 32) conducted their baseline and endline surveys during distinct transmission season what also could have led to the introduction of bias into the results. It cannot be ruled out that some studies have used climate information to correct for changes in control and treatment groups that were unrelated to the interventions. Some studies measured very different baseline data between intervention and control group, adding to the list of methodological problems. Another point is that the studies used different container types. It can be reasoned that they use the kind of containers that are locally available to be representative, but it also leads to incomparability between the studies. Only four (14, 27, 31, 32) of 19 studies had an untreated control group. This can also result in difficulties of comparability.

Most studies implemented multiple environmental vector control methods, making it difficult to measure the effect of an individual method. On the other hand, a combination of multiple methods is necessary, particularly targeting larvae and adult mosquitoes, since this could increase the success in reducing vector indices. This could be a reason why our study could not identify obvious differences in the effectiveness of the different vector control methods. The mode of delivery of interventions could also influence the effectiveness, the community-based or the vector control services [36].

Most studies are not comparable because of the high variability among the studies. With the multitude of methodological issues, perhaps a systematic review makes more sense than a meta-analysis. This limits the possibility for meta-analysis also considering that for example effects of clustering for the variability of data for the main figures cannot be easily addressed. In any case, consistent protocols and better standards for vector control studies are strongly needed. Additionally, an ongoing engagement of both, the communities and public health experts is required, regarding the long-term effectiveness of interventions. As a further limitation of this systematic review and meta-analysis our searches were finished in May 2017. However, according to our knowledge no further studies have been published that would have altered our results nor several overviews have been published.

In summary, regarding both, systematic review and meta-analysis, all environmental dengue vector control interventions showed some effectiveness reducing larval and pupal densities of *Aedes* sp. mosquitoes. However, there is a strong need for more comparable high-quality studies at an adequate standard to strengthen this evidence. Efforts should be directed at the creation of consistent study protocols, including (i) the creation of guidelines about the maximum distortions of baseline data and housing situations between intervention and control clusters, (ii) sustainability and cost effectiveness and (iii) community acceptance.

## Supporting information

S1 Appendix Literature search terms for all databases. (PDF)

S2 Appendix R script. (PDF)

S3 Appendix Quality assessment. (PDF)

S4 Appendix PRISMA 2009 checklist. (PDF)

S5 Appendix PRISMA 2009 flow diagram. (PDF)

S6 Appendix Formulas for difference-in-differences and difference-of-endlines calculation. (PDF)

S1 Table Evidence table. (PDF)

S2 Table Meta-analysis data of container covers without insecticides for the outcome measure BI. (TIF)

S3 Table Meta-analysis data of container covers without insecticides for the outcome measure PPI. (TIF)

S4 Table Meta-analysis data of container covers with insecticides for the outcome measure BI. (TIF)

S5 Table Meta-analysis data of container covers with insecticides for the outcome measure PPI. (TIF)

S6 Table Meta-analysis data of waste management with direct garbage collection for the outcome measure BI. (TIF)

S7 Table Meta-analysis data of waste management with direct garbage collection for the outcome measure PPI. (TIF)

S8 Table Meta-analysis data of waste management without direct garbage collection for the outcome measure BI. (TIF)

S9 Table Meta-analysis data of waste management without direct garbage collection for the outcome measure PPI. (TIF)

S10 Table Meta-analysis data of elimination of breeding places for the outcome measure BI. (TIF)

S11 Table Meta-analysis data of elimination of breeding places for the outcome measure PPI. (TIF)

S1 Fig Forest Plot of the pooled estimates for the outcome Breteau Index (different scaling). (TIF)

S2 Fig Forest Plot of the pooled estimates for the outcome Pupae per Person Index (different scaling). (TIF)
