# Supplementary material for: Environmental methods for dengue vector control – A systematic review and meta-analysis
Source: PLoS Negl Trop Dis. 2019 Jul 11;13(7):e0007420. doi: 10.1371/journal.pntd.0007420 (PMC6650086; doi:10.1371/journal.pntd.0007420)
Supplement: S1 Appendix — (PDF) [file pntd.0007420.s001.pdf]

S1 Appendix. Literature search terms for all databases

PubMed

Number	Search terms
**1**	dengue environment vector control
**2**	dengue container
**3**	dengue waste
**4**	dengue clean
**5**	dengue source reduction
**6**	dengue breeding AND (reduction OR elimination)
**7**	dengue water storage

EMBASE

Number	Search terms
**1**	dengue AND environment AND vector AND control
**2**	dengue AND container
**3**	dengue AND waste
**4**	dengue AND clean
**5**	dengue AND source AND reduction
**6**	dengue AND breeding AND (reduction OR elimination)
**7**	dengue AND water AND storage

LILACS

Number	Search terms
**1**	dengue environment vector control
**2**	dengue container
**3**	dengue waste
**4**	dengue clean
**5**	dengue source reduction
**6**	(dengue breeding reduction) OR (dengue breeding elimination) OR (dengue breeding reduction elimination)
**7**	dengue water storage

WHOLIS

Number	Search terms
**1**	dengue AND control
**2**	dengue AND management
**3**	dengue AND environment

The Cochrane Library

Number	Search terms
**1**	dengue environment vector control
**2**	dengue container
**3**	dengue waste
**4**	dengue clean
**5**	dengue source reduction
**6**	(dengue breeding reduction) OR (dengue breeding elimination) OR (dengue breeding reduction elimination)
**7**	dengue water storage

Google Scholar

Number	Search terms
**1**	dengue environment vector control
**2**	dengue container cover
**3**	dengue clean-up campaign
**4**	dengue waste management
**5**	dengue elimination of breeding sites aedes
**6**	Dengue vector source reduction
**7**	dengue water storage practices

PubMed search results

1. search term	2. search term	3. search term	Hits	Records screened
dengue	environment	vector control	676	43
dengue	container		277	41
dengue	waste		62	16
dengue	clean		36	12
dengue	source reduction		81	17
dengue	breeding	Reduction OR elimination	99	16
dengue	water storage		46	23
