# Supplementary material for: Environmental methods for dengue vector control – A systematic review and meta-analysis
Source: PLoS Negl Trop Dis. 2019 Jul 11;13(7):e0007420. doi: 10.1371/journal.pntd.0007420 (PMC6650086; doi:10.1371/journal.pntd.0007420)
Supplement: S2 Appendix — (PDF) [file pntd.0007420.s002.pdf]

S2 Appendix. R script

```r
library(forestplot)
library(rmeta)
library(Gmisc)
library(xlsx)
library(readxl)

#Container covers without insecticides - Breteau Index

#Generation of matrices including study group, n, BI baseline, BI endline
#and the coefficients and intervals of difference-in-differences and difference-of-endlines.

BICCdiffdiffwithout <-
  structure(
    c(1, 0, 1, 0, 1, 0, 1, 0, 1, 0, NA,
      NA, NA, 1000, 1000, 1689, 1580, 1000, 1000, 441, 448, NA,
      20, 37.5, 3.4, 2.64, 1.5991, 1.0278, 30.8, 22.2, 81.86, 78.79, NA,
      10.8, 46.9, 12.02, 13.77, 2.4646, 4.3158, 4.3, 21.4, 24.46, 21.49, NA,
      -18.6, NA, -2.51, NA, -2.4225, NA, -25.7, NA, -0.1, NA, -7.8975,
      -18.6, NA, -2.51, NA, -2.4225, NA, -25.7, NA, -0.1, NA, -7.8975,
      -18.6, NA, -2.51, NA, -2.4225, NA, -25.7, NA, -0.1, NA, -7.8975),
    .Dim = c(11L, 7L),
    .Dimnames = list(c("Overgaard", "2016",
                       "Basso", "2015",
                       "Caprara", "2015",
                       "Arunachalam", "2012",
                       "Kittayapong", "2012",
                       "Pooled"),
    c("group", "n", "BI (b)", "BI (e)", "coef", "lower", "upper"))
  )

BICCdiffendlwithout <-
  structure(
    c(1, 0, 1, 0, 1, 0, 1, 0, 1, 0, NA,
      NA, NA, 1000, 1000, 1689, 1580, 1000, 1000, 441, 448, NA,
      20, 37.5, 3.4, 2.64, 1.5991, 1.0278, 30.8, 22.2, 81.86, 78.79, NA,
      10.8, 46.9, 12.02, 13.77, 2.4646, 4.3158, 4.3, 21.4, 24.46, 21.49, NA,
      -36.1, NA, -1.75, NA, -1.85, NA, -17.1, NA, 2.97, NA, -5.0394,
      -36.1, NA, -1.75, NA, -1.85, NA, -17.1, NA, 2.97, NA, -5.0394,
      -36.1, NA, -1.75, NA, -1.85, NA, -17.1, NA, 2.97, NA, -5.0394),
    .Dim = c(11L, 7L),
    .Dimnames = list(c("Overgaard", "2016",
                       "Basso", "2015",
                       "Caprara", "2015",
                       "Arunachalam", "2012",
                       "Kittayapong", "2012",
                       "Pooled"),
                     c("group", "n", "BI (b)", "BI (e)", "coef", "lower", "upper"))
  )

#Introduction of a header for the columns

BICCdiffdiffheader <- rbind(rep(NA,times=ncol(BICCdiffdiffwithout)),
                    BICCdiffdiffwithout)
rownames(BICCdiffdiffheader)[1] <- "Author"
BICCdiffendlheader <- rbind(rep(NA,times=ncol(BICCdiffendlwithout)),
                            BICCdiffendlwithout)
rownames(BICCdiffendlheader)[1] <- "Author"

#Generation of a label matrix including group, n, BI baseline, BI endline,
#difference-in-differences and difference-of-endlines.

lab_mtrx_CCBI <- cbind(rownames(BICCdiffdiffheader),
                    append("group",
                           round(BICCdiffdiffheader[,"group"],0)[2:12]),
                    append("n",
                           round(BICCdiffdiffheader[,"n"],0)[2:12]),
                    append("BI (b)",
                           round(BICCdiffdiffheader[,"BI (b)"],1)[2:12]),
                    append("BI (e)",
                           round(BICCdiffdiffheader[,"BI (e)"],1)[2:12]),
                    append("DID",
                           round(BICCdiffdiffheader[,"coef"],1)[2:12]),
                    append("DOE",
```

```
                          round(BICCdiffendlheader[,"coef"],1)[2:12]))

#Generation of an excel table which was manually processed and red in R again

write.xlsx(lab_mtrx_CCBI, "C:/Users/Claudia/Desktop/R_Plot/lab_mtrx_CCBI.xlsx")
lab_mtrx_CCBI <- read_excel("C:/Users/Claudia/Desktop/R_Plot/lab_mtrx_CCBIv2.xlsx")

#Generation of the forest plot with label matrix

forestplot2(mean=cbind(BICCdiffdiffheader[,"coef"], BICCdiffendlheader[,"coef"]),
            lower=cbind(BICCdiffdiffheader[,"lower"], BICCdiffendlheader[,"lower"]),
            upper=cbind(BICCdiffdiffheader[,"upper"], BICCdiffendlheader[,"upper"]),
            labeltext=lab_mtrx_CCBI,
            txt_gp = fpTxtGp(label = gpar(cex = 0.7),
                             legend = gpar(cex = 0.7),
                             ticks = gpar(cex = 0.7)),
            is.summary=c(TRUE, rep(FALSE, times=nrow(BICCdiffdiffheader)-1)),
            clip=c(-40, 5),
            xticks=c(-40, -30, -20, -10, 0, 10),
            boxsize=
                 c(0.0,
                   0.6,
                   0,
                   0.35,
                   0,
                   0.44,
                   0,
                   0.35,
                   0,
                   0.27,
                   0,
                   1),
            col=fpColors(box=c("darkred","darkblue")),
            fn.ci_norm=
              list(fpDrawCircleCI,
                   fpDrawCircleCI,
                   fpDrawNormalCI,
                   fpDrawNormalCI,
                   fpDrawNormalCI,
                   fpDrawNormalCI,
                   fpDrawNormalCI,
                   fpDrawNormalCI,
                   fpDrawNormalCI,
                   fpDrawNormalCI,
                   fpDrawNormalCI,
                   fpDrawDiamondCI),
            pch=13,
            new_page=TRUE,
            hrzl_lines = list("4" = gpar(lty=2),
                              "2" = gpar(col="#444444"),
                              "6" = gpar(col="#444444"),
                              "8" = gpar(col="#444444"),
                              "10" = gpar(col="#444444"),
                              "12" = gpar(col="#444444")),
            colgap = unit(5, "mm"),
            lineheight = unit(0.6, "cm"),
            line.margin = unit(0.2, "cm"),
            graphwidth = unit(10, "cm"),
            legend=c("difference-in-differences (DID)","difference-of-endlines (DOE)"),
            legend_args = fpLegend(pos = list(x=0.0, y=-0.2))
)

#Container covers without insecticides - Pupae Per Person Index

#Generation of matrices including study group, n, BI baseline, BI endline
#and the coefficients and intervals of difference-in-differences and difference-of-endlines.

PPICCdiffdiffwithout <-
  structure(
    c(1, 0, 1, 0, 1, 0, 1, 0, 1, 0, 1, 0, NA,
      NA, NA, 2000, 1000, 1689, 1580, 1000, 1000, 441, 448, 592, 600, NA,
      0.0, 0.06, 0.048, 0.016, 0.0229, 0.0104, 1.075, 0.729, 0.44, 0.42, 0.34, 0.33, NA,
      0.04, 0.36, 0.13, 0.14, 0.0292, 0.0539, 0.004, 0.355, 0.04, 0.4, 0.23, 0.15, NA,
      -0.26, NA, -0.042, NA, -0.0372, NA, -0.697, NA, -0.38, NA, 0.07, NA, -0.1831,
      -0.26, NA, -0.042, NA, -0.0372, NA, -0.697, NA, -0.38, NA, 0.07, NA, -0.1831,
      -0.26, NA, -0.042, NA, -0.0372, NA, -0.697, NA, -0.38, NA, 0.07, NA, -0.1831),
    .Dim = c(13L, 7L),
    .Dimnames = list(c("Overgaard", "2016",
```

```
                          "Basso", "2015",
                          "Caprara", "2015",
                          "Arunachalam", "2012",
                          "Kittayapong", "2012",
                          "Wai", "2012",
                          "Pooled"),
                        c("group", "n", "PPI (b)", "PPI (e)", "coef", "lower", "upper"))
  )

PPICCdiffendlwithout <-
  structure(
    c(1, 0, 1, 0, 1, 0, 1, 0, 1, 0, 1, 0, NA,
      NA, NA, 2000, 1000, 1689, 1580, 1000, 1000, 441, 448, 592, 600, NA,
      0.0, 0.06, 0.048, 0.016, 0.0229, 0.0104, 1.075, 0.729, 0.44, 0.42, 0.34, 0.33, NA,
      0.04, 0.36, 0.13, 0.14, 0.0292, 0.0539, 0.004, 0.355, 0.04, 0.4, 0.23, 0.15, NA,
      -0.32, NA, -0.01, NA, -0.02, NA, -0.35, NA, -0.36, NA, 0.08, NA, -0.1000,
      -0.32, NA, -0.01, NA, -0.02, NA, -0.35, NA, -0.36, NA, 0.08, NA, -0.1000,
      -0.32, NA, -0.01, NA, -0.02, NA, -0.35, NA, -0.36, NA, 0.08, NA, -0.1000),
    .Dim = c(13L, 7L),
    .Dimnames = list(c("Overgaard", "2016",
                          "Basso", "2015",
                          "Caprara", "2015",
                          "Arunachalam", "2012",
                          "Kittayapong", "2012",
                          "Wai", "2012",
                          "Pooled"),
                        c("group", "n", "PPI (b)", "PPI (e)", "coef", "lower", "upper"))
  )

#Introduction of a header for the columns

PPICCdiffdiffheader <- rbind(rep(NA,times=ncol(PPICCdiffdiffwithout)),
                             PPICCdiffdiffwithout)
PPICCdiffendlheader <- rbind(rep(NA,times=ncol(PPICCdiffendlwithout)),
                              PPICCdiffendlwithout)
rownames(PPICCdiffdiffheader)[1] <- "Author"
rownames(PPICCdiffendlheader)[1] <- "Author"

#Generation of a label matrix including group, n, BI baseline, BI endline,
#difference-in-differences and difference-of-endlines.

lab_mtrx_CCPPI <- cbind(rownames(PPICCdiffdiffheader),
                        append("group",
                               round(PPICCdiffdiffheader[,"group"],0)[2:14]),
                        append("n",
                               round(PPICCdiffdiffheader[,"n"],0)[2:14]),
                        append("PPI (b)",
                               round(PPICCdiffdiffheader[,"PPI (b)"],2)[2:14]),
                        append("PPI (e)",
                               round(PPICCdiffdiffheader[,"PPI (e)"],2)[2:14]),
                        append("DID",
                               round(PPICCdiffdiffheader[,"coef"],2)[2:14]),
                        append("DOE",
                               round(PPICCdiffendlheader[,"coef"],2)[2:14]))

#Generation of an excel table which was manually processed and red in R again

write.xlsx(lab_mtrx_CCPPI, "C:/Users/Claudia/Desktop/R_Plot/lab_mtrx_CCPPI.xlsx")
lab_mtrx_CCPPI <- read_excel("C:/Users/Claudia/Desktop/R_Plot/lab_mtrx_CCPPIv2.xlsx")

#Generation of the forest plot with label matrix

forestplot2(mean=cbind(PPICCdiffdiffheader[,"coef"], PPICCdiffendlheader[,"coef"]),
            lower=cbind(PPICCdiffdiffheader[,"lower"], PPICCdiffendlheader[,"lower"]),
            upper=cbind(PPICCdiffdiffheader[,"upper"], PPICCdiffendlheader[,"upper"]),
            labeltext=lab_mtrx_CCPPI,
            txt_gp = fpTxtGp(label = gpar(cex = 0.7),
                              legend = gpar(cex = 0.7),
                              ticks = gpar(cex = 0.7)),
            is.summary=c(TRUE, rep(FALSE, times=nrow(PPICCdiffdiffheader)-1)),
            clip=c(-1, 0.5),
            xticks=c(-1, -0.5, 0, 0.5),
            boxsize=
              c(0.0,
                0.6,
                0,
                0.37,
                0,
```

```
                0.39,
                0,
                0.32,
                0,
                0.25,
                0,
                0.27,
                0,
                1),
            col=fpColors(box=c("darkred", "darkblue")),
            fn.ci_norm=
              list(fpDrawCircleCI,
                   fpDrawCircleCI,
                   fpDrawNormalCI,
                   fpDrawNormalCI,
                   fpDrawNormalCI,
                   fpDrawNormalCI,
                   fpDrawNormalCI,
                   fpDrawNormalCI,
                   fpDrawNormalCI,
                   fpDrawNormalCI,
                   fpDrawNormalCI,
                   fpDrawNormalCI,
                   fpDrawNormalCI,
                   fpDrawDiamondCI),
            pch=13,
            new_page=TRUE,
            hrzl_lines = list("4" = gpar(lty=2),
                              "2" = gpar(col="#444444"),
                              "6" = gpar(col="#444444"),
                              "8" = gpar(col="#444444"),
                              "10" = gpar(col="#444444"),
                              "12" = gpar(col="#444444"),
                              "14" = gpar(col="#444444")),
            colgap = unit(5, "mm"),
            lineheight = unit(0.6, "cm"),
            line.margin = unit(0.2, "cm"),
            graphwidth = unit(10, "cm"),
            legend=c("difference-in-differences (DID)","difference-of-endlines (DOE)"),
            legend_args = fpLegend(pos = list(x=0.0, y=-0.2))
)

#Container covers with insecticides - Breteau Index

#Generation of matrices including study group, n, BI baseline, BI endline
#and the coefficients and intervals of difference-in-differences and difference-of-endlines.

BICCdiffdiffwith <-
  structure(
    c(1, 0, 1, 0, 1, 0, 1, 0, NA,
      549, 549, 327, 282, 720, 720, 558, 558, NA,
      60, 113, 8.9, 5, 11.2, 6.6, 38, 34, NA,
      7, 12, 4.5, 3.2, 14.1, 8.6, 11, 17, NA,
      48, NA, -2.6, NA, 0.9, NA, -10, NA, -3.616872038,
      48, NA, -2.6, NA, 0.9, NA, -10, NA, -3.616872038,
      48, NA, -2.6, NA, 0.9, NA, -10, NA, -3.616872038),
    .Dim = c(9L, 7L),
    .Dimnames = list(c("Kroeger (Mexico)", "2006",
                       "Quintero", "2015",
                       "Tun-Lin (Venezuela)", "2009",
                       "Kroeger (Venezuela)", "2006",
                       "Pooled"),
                     c("group", "n", "BI (b)", "BI (e)", "coef", "lower", "upper"))
  )

BICCdiffendlwith <-
  structure(
    c(1, 0, 1, 0, 1, 0, 1, 0, NA,
      549, 549, 327, 282, 720, 720, 558, 558, NA,
      60, 113, 8.9, 5, 11.2, 6.6, 38, 34, NA,
      7, 12, 4.5, 3.2, 14.1, 8.6, 11, 17, NA,
      -5, NA, 1.3, NA, 5.5, NA, -6, NA, 0.63687204,
      -5, NA, 1.3, NA, 5.5, NA, -6, NA, 0.63687204,
      -5, NA, 1.3, NA, 5.5, NA, -6, NA, 0.63687204),
    .Dim = c(9L, 7L),
    .Dimnames = list(c("Kroeger (Mexico)", "2006",
                       "Quintero", "2015",
                       "Tun-Lin (Venezuela)", "2009",
```

```r
                         "Kroeger (Venezuela)", "2006",
                         "Pooled"),
                       c("group", "n", "BI (b)", "BI (e)", "coef", "lower", "upper"))
  )

#Introduction of a header for the columns

BICCdiffdiffwithheader <- rbind(rep(NA,times=ncol(BICCdiffdiffwith)),
                            BICCdiffdiffwith)
rownames(BICCdiffdiffwithheader)[1] <- "Author"
BICCdiffendlwithheader <- rbind(rep(NA,times=ncol(BICCdiffendlwith)),
                            BICCdiffendlwith)
rownames(BICCdiffendlwithheader)[1] <- "Author"

#Generation of a label matrix including group, n, BI baseline, BI endline,
#difference-in-differences and difference-of-endlines.

lab_mtrx_CCBIwith <- cbind(rownames(BICCdiffdiffwithheader),
                    append("group",
                           round(BICCdiffdiffwithheader[,"group"],0)[2:10]),
                    append("n",
                           round(BICCdiffdiffwithheader[,"n"],0)[2:10]),
                    append("BI (b)",
                           round(BICCdiffdiffwithheader[,"BI (b)"],1)[2:10]),
                    append("BI (e)",
                           round(BICCdiffdiffwithheader[,"BI (e)"],1)[2:10]),
                    append("DID",
                           round(BICCdiffdiffwithheader[,"coef"],1)[2:10]),
                    append("DOE",
                           round(BICCdiffendlwithheader[,"coef"],1)[2:10]))

#Generation of an excel table which was manually processed and red in R again

write.xlsx(lab_mtrx_CCBIwith, "C:/Users/Claudia/Desktop/R_Plot/lab_mtrx_CCBIwith.xlsx")
lab_mtrx_CCBIwith <- read_excel("C:/Users/Claudia/Desktop/R_Plot/lab_mtrx_CCBIwithv2.xlsx")

#Generation of the forest plot with label matrix

forestplot2(mean=cbind(BICCdiffdiffwithheader[,"coef"], BICCdiffendlwithheader[,"coef"]),
            lower=cbind(BICCdiffdiffwithheader[,"lower"], BICCdiffendlwithheader[,"lower"]),
            upper=cbind(BICCdiffdiffwithheader[,"upper"], BICCdiffendlwithheader[,"upper"]),
            labeltext=lab_mtrx_CCBIwith,
            txt_gp = fpTxtGp(label = gpar(cex = 0.7),
                             legend = gpar(cex = 0.7),
                             ticks = gpar(cex = 0.7)),
            is.summary=c(TRUE, rep(FALSE, times=nrow(BICCdiffdiffwithheader)-1)),
            clip=c(-20, 50),
            xticks=c(-20, -10, 0, 10, 20, 30, 40, 50),
            boxsize=
              c(0.0,
                0.6,
                0,
                0.32,
                0,
                0.47,
                0,
                0.41,
                0,
                1),
            col=fpColors(box=c("darkred","darkblue")),
            fn.ci_norm=
              list(fpDrawCircleCI,
                   fpDrawCircleCI,
                   fpDrawNormalCI,
                   fpDrawNormalCI,
                   fpDrawNormalCI,
                   fpDrawNormalCI,
                   fpDrawNormalCI,
                   fpDrawNormalCI,
                   fpDrawNormalCI,
                   fpDrawDiamondCI),
            pch=13,
            new_page=TRUE,
            hrzl_lines = list("2" = gpar(col="#444444"),
                              "4" = gpar(lty=2),
                              "6" = gpar(col="#444444"),
                              "8" = gpar(col="#444444"),
                              "10" = gpar(col="#444444")),
```

```
            colgap = unit(5, "mm"),
            lineheight = unit(0.6, "cm"),
            line.margin = unit(0.2, "cm"),
            graphwidth = unit(10, "cm"),
            legend=c("difference-in-differences (DID)","difference-of-endlines (DOE)"),
            legend_args = fpLegend(pos = list(x=0.0, y=-0.25))
)

#Container covers with insecticides - Pupae Per Person Index

#Generation of matrices including study group, n, BI baseline, BI endline
#and the coefficients and intervals of difference-in-differences and difference-of-endlines.

PPICCdiffdiffwith <-
  structure(
    c(1, 0, 1, 0, 1, 0, 1, 0, NA,
      327, 282, 720, 720, 549, 549, 558, 558, NA,
      1.25, 0.24, 0.61, 0.23, 3.4, 2, 2.7, 1.6, NA,
      0.23, 0.32, 0.57, 0.2, 0.36, 0.35, 0.2, 0.3, NA,
      -1.1, NA, -0.01, NA, -1.39, NA, -1.2, NA, -0.833,
      -1.1, NA, -0.01, NA, -1.39, NA, -1.2, NA, -0.833,
      -1.1, NA, -0.01, NA, -1.39, NA, -1.2, NA, -0.833),
    .Dim = c(9L, 7L),
    .Dimnames = list(c("Quintero", "2015",
                       "Tun-Lin (Venezuela)", "2009",
                       "Kroeger (Mexico)", "2006",
                       "Kroeger (Venezuela)", "2006",
                       "Pooled"),
                     c("group", "n", "PPI (b)", "PPI (e)", "coef", "lower", "upper"))
  )

PPICCdiffendlwith <-
  structure(
    c(1, 0, 1, 0, 1, 0, 1, 0, NA,
      327, 282, 720, 720, 549, 549, 558, 558, NA,
      1.25, 0.24, 0.61, 0.23, 3.4, 2, 2.7, 1.6, NA,
      0.23, 0.32, 0.57, 0.2, 0.36, 0.35, 0.2, 0.3, NA,
      -0.09, NA, 0.37, NA, 0.01, NA, -0.1, NA, 0.086,
      -0.09, NA, 0.37, NA, 0.01, NA, -0.1, NA, 0.086,
      -0.09, NA, 0.37, NA, 0.01, NA, -0.1, NA, 0.086),
    .Dim = c(9L, 7L),
    .Dimnames = list(c("Quintero", "2015",
                       "Tun-Lin (Venezuela)", "2009",
                       "Kroeger (Mexico)", "2006",
                       "Kroeger (Venezuela)", "2006",
                       "Pooled"),
                     c("group", "n", "PPI (b)", "PPI (e)", "coef", "lower", "upper"))
  )

#Introduction of a header for the columns

PPICCdiffdiffwithheader <- rbind(rep(NA,times=ncol(PPICCdiffdiffwith)),
                                PPICCdiffdiffwith)
rownames(PPICCdiffdiffwithheader)[1] <- "Author"
PPICCdiffendlwithheader <- rbind(rep(NA,times=ncol(PPICCdiffendlwith)),
                                PPICCdiffendlwith)
rownames(PPICCdiffendlwithheader)[1] <- "Author"

#Generation of a label matrix including group, n, BI baseline, BI endline,
#difference-in-differences and difference-of-endlines.

lab_mtrx_CCPPIwith <- cbind(rownames(PPICCdiffdiffwithheader),
                           append("group",
                                  round(PPICCdiffdiffwithheader[,"group"],0)[2:10]),
                           append("n",
                                  round(PPICCdiffdiffwithheader[,"n"],0)[2:10]),
                           append("PPI (b)",
                                  round(PPICCdiffdiffwithheader[,"PPI (b)"],2)[2:10]),
                           append("PPI (e)",
                                  round(PPICCdiffdiffwithheader[,"PPI (e)"],2)[2:10]),
                           append("DID",
                                  round(PPICCdiffdiffwithheader[,"coef"],2)[2:10]),
                           append("DOE",
                                  round(PPICCdiffendlwithheader[,"coef"],2)[2:10]))

#Generation of an excel table which was manually processed and red in R again

write.xlsx(lab_mtrx_CCPPIwith, "C:/Users/Claudia/Desktop/R_Plot/lab_mtrx_CCPPIwith.xlsx")
```

```
lab_mtrx_CCPPIwith <- read_excel("C:/Users/Claudia/Desktop/R_Plot/lab_mtrx_CCPPIwithv2.xlsx")

#Generation of the forest plot with label matrix

forestplot2(mean=cbind(PPICCdiffdiffwithheader[,"coef"], PPICCdiffendlwithheader[,"coef"]),
            lower=cbind(PPICCdiffdiffwithheader[,"lower"], PPICCdiffendlwithheader[,"lower"]),
            upper=cbind(PPICCdiffdiffwithheader[,"upper"], PPICCdiffendlwithheader[,"upper"]),
            labeltext=lab_mtrx_CCPPIwith,
            txt_gp = fpTxtGp(label = gpar(cex = 0.7),
                             legend = gpar(cex = 0.7),
                             ticks = gpar(cex = 0.7)),
            is.summary=c(TRUE, rep(FALSE, times=nrow(PPICCdiffdiffwithheader)-1)),
            clip=c(-2, 0.5),
            xticks=c(-2, -1.5, -1, -0.5, 0, 0.5),
            boxsize=
              c(0.0,
                0.29,
                0,
                0.40,
                0,
                0.35,
                0,
                0.36,
                0,
                1),
            col=fpColors(box=c("darkred","darkblue")),
            fn.ci_norm=
              list(fpDrawNormalCI,
                   fpDrawNormalCI,
                   fpDrawNormalCI,
                   fpDrawNormalCI,
                   fpDrawNormalCI,
                   fpDrawNormalCI,
                   fpDrawNormalCI,
                   fpDrawNormalCI,
                   fpDrawNormalCI,
                   fpDrawDiamondCI),
            pch=13,
            new_page=TRUE,
            hrzl_lines = list("2" = gpar(col="#444444"),
                              "4" = gpar(col="#444444"),
                              "6" = gpar(col="#444444"),
                              "8" = gpar(col="#444444"),
                              "10" = gpar(col="#444444")),
            colgap = unit(5, "mm"),
            lineheight = unit(0.6, "cm"),
            line.margin = unit(0.2, "cm"),
            graphwidth = unit(10, "cm"),
            legend=c("difference-in-differences (DID)","difference-of-endlines (DOE)"),
            legend_args = fpLegend(pos = list(x=0.0, y=-0.25))
)

#Waste management without direct garbage collection - Breteau Index

#Generation of matrices including study group, n, BI baseline, BI endline
#and the coefficients and intervals of difference-in-differences and difference-of-endlines.

BIWMdiffdiffwithout <-
  structure(
    c(1, 0, 1, 0, 1, 0, 1, 0, NA,
      9529, 9309, 200, 200, 8422, 10748, 225, 225, NA,
      NA, NA, 6.9, 2, 0.27, 0.2, 41.5, 35, NA,
      19.7, 30.2, 0.5, 3.5, 0.28, 0.52, 34, 46, NA,
      NA, NA, -7.9, NA, -0.31, NA, -18.5, NA, -0.448,
      NA, NA, -7.9, NA, -0.31, NA, -18.5, NA, -0.448,
      NA, NA, -7.9, NA, -0.31, NA, -18.5, NA, -0.448),
    .Dim = c(9L, 7L),
    .Dimnames = list(c("Andersson", "2015",
                       "Tana", "2012",
                       "Vanlerberghe", "2009",
                       "Leontsini", "1993",
                       "Pooled"),
                     c("group", "n", "BI (b)", "BI (e)", "coef", "lower", "upper"))
  )

BIWMdiffendlwithout <-
  structure(
    c(1, 0, 1, 0, 1, 0, 1, 0, NA,
```

```
      9529, 9309, 200, 200, 8422, 10748, 225, 225, NA,
      NA, NA, 6.9, 2, 0.27, 0.2, 41.5, 35, NA,
      19.7, 30.2, 0.5, 3.5, 0.28, 0.52, 34, 46, NA,
      -10.5, NA, -3, NA, -0.24, NA, -12, NA, -5.379,
      -10.5, NA, -3, NA, -0.24, NA, -12, NA, -5.379,
      -10.5, NA, -3, NA, -0.24, NA, -12, NA, -5.379),
    .Dim = c(9L, 7L),
    .Dimnames = list(c("Andersson", "2015",
                       "Tana", "2012",
                       "Vanlerberghe", "2009",
                       "Leontsini", "1993",
                       "Pooled"),
                     c("group", "n", "BI (b)", "BI (e)", "coef", "lower", "upper"))
  )

#Introduction of a header for the columns

BIWMdiffdiffheader <- rbind(rep(NA,times=ncol(BIWMdiffdiffwithout)),
                            BIWMdiffdiffwithout)
rownames(BIWMdiffdiffheader)[1] <- "Author"
BIWMdiffendlheader <- rbind(rep(NA,times=ncol(BIWMdiffendlwithout)),
                            BIWMdiffendlwithout)
rownames(BIWMdiffendlheader)[1] <- "Author"

#Generation of a label matrix including group, n, BI baseline, BI endline,
#difference-in-differences and difference-of-endlines.

lab_mtrx_WMBI <- cbind(rownames(BIWMdiffdiffheader),
                       append("group",
                              round(BIWMdiffdiffheader[,"group"],0)[2:10]),
                       append("n",
                              round(BIWMdiffdiffheader[,"n"],0)[2:10]),
                       append("BI (b)",
                              round(BIWMdiffdiffheader[,"BI (b)"],1)[2:10]),
                       append("BI (e)",
                              round(BIWMdiffdiffheader[,"BI (e)"],1)[2:10]),
                       append("DID",
                              round(BIWMdiffdiffheader[,"coef"],1)[2:10]),
                       append("DOE",
                              round(BIWMdiffendlheader[,"coef"],1)[2:10]))

#Generation of an excel table which was manually processed and red in R again

write.xlsx(lab_mtrx_WMBI, "C:/Users/Claudia/Desktop/R_Plot/lab_mtrx_WMBI.xlsx")
lab_mtrx_WMBI <- read_excel("C:/Users/Claudia/Desktop/R_Plot/lab_mtrx_WMBIv2.xlsx")

#Generation of the forest plot with label matrix

forestplot2(mean=cbind(BIWMdiffdiffheader[,"coef"], BIWMdiffendlheader[,"coef"]),
            lower=cbind(BIWMdiffdiffheader[,"lower"], BIWMdiffendlheader[,"lower"]),
            upper=cbind(BIWMdiffdiffheader[,"upper"], BIWMdiffendlheader[,"upper"]),
            labeltext=lab_mtrx_WMBI,
            txt_gp = fpTxtGp(label = gpar(cex = 0.7),
                             legend = gpar(cex = 0.7),
                             ticks = gpar(cex = 0.7)),
            is.summary=c(TRUE, rep(FALSE, times=nrow(BIWMdiffdiffheader)-1)),
            clip=c(-20, 5),
            xticks=c(-20, -15, -10, -5, 0, 5),
            boxsize=
              c(0.0,
                0.49,
                0,
                0.21,
                0,
                0.5,
                0,
                0.21,
                0,
                1),
            col=fpColors(box=c("darkred","darkblue")),
            fn.ci_norm=
              list(fpDrawNormalCI,
                   fpDrawNormalCI,
                   fpDrawNormalCI,
                   fpDrawNormalCI,
                   fpDrawNormalCI,
                   fpDrawNormalCI,
                   fpDrawNormalCI,
```

```
                    fpDrawNormalCI,
                    fpDrawNormalCI,
                    fpDrawDiamondCI),
            pch=13,
            new_page=TRUE,
            hrzl_lines = list("2" = gpar(col="#444444"),
                              "4" = gpar(col="#444444"),
                              "6" = gpar(col="#444444"),
                              "8" = gpar(col="#444444"),
                              "10" = gpar(col="#444444")),
            colgap = unit(5, "mm"),
            lineheight = unit(0.6, "cm"),
            line.margin = unit(0.2, "cm"),
            graphwidth = unit(10, "cm"),
            legend=c("difference-in-differences (DID)","difference-of-endlines (DOE)"),
            legend_args = fpLegend(pos = list(x=0.0, y=-0.25))
)

#Waste management without direct garbage collection - Pupae Per Person Index

#Generation of matrices including study group, n, BI baseline, BI endline
#and the coefficients and intervals of difference-in-differences and difference-of-endlines.

PPIWMdiffdiffwithout <-
  structure(
    c(1, 0, 1, 0, 1, 0, 1, 0, NA,
      9529, 9309, 1000, 1000, 200, 200, 8422, 10748, NA,
      NA, NA, 0.524, 0.817, 0.015, 0.05, 0.44, 0.29, NA,
      0.092, 0.175, 0.08, 0.353, 0.005, 0.04, 0.36, 1.4, NA,
      NA, NA, 0.02, NA, 0, NA, -1.19, NA, -0.564,
      NA, NA, 0.02, NA, 0, NA, -1.19, NA, -0.564,
      NA, NA, 0.02, NA, 0, NA, -1.19, NA, -0.564),
    .Dim = c(9L, 7L),
    .Dimnames = list(c("Andersson", "2015",
                       "Mitchell-Foster", "2015",
                       "Tana", "2012",
                       "Vanlerberghe", "2009",
                       "Pooled"),
                     c("group", "n", "PPI (b)", "PPI (e)", "coef", "lower", "upper"))
  )

PPIWMdiffendlwithout <-
  structure(
    c(1, 0, 1, 0, 1, 0, 1, 0, NA,
      9529, 9309, 1000, 1000, 200, 200, 8422, 10748, NA,
      NA, NA, 0.524, 0.817, 0.015, 0.05, 0.44, 0.29, NA,
      0.092, 0.175, 0.08, 0.353, 0.005, 0.04, 0.36, 1.4, NA,
      -0.083, NA, -0.273, NA, -0.035, NA, -1.04, NA, -0.546,
      -0.083, NA, -0.273, NA, -0.035, NA, -1.04, NA, -0.546,
      -0.083, NA, -0.273, NA, -0.035, NA, -1.04, NA, -0.546),
    .Dim = c(9L, 7L),
    .Dimnames = list(c("Andersson", "2015",
                       "Mitchell-Foster", "2015",
                       "Tana", "2012",
                       "Vanlerberghe", "2009",
                       "Pooled"),
                     c("group", "n", "PPI (b)", "PPI (e)", "coef", "lower", "upper"))
  )

#Introduction of a header for the columns

PPIWMdiffdiffheader <- rbind(rep(NA,times=ncol(PPIWMdiffdiffwithout)),
                             PPIWMdiffdiffwithout)
rownames(PPIWMdiffdiffheader)[1] <- "Author"
PPIWMdiffendlheader <- rbind(rep(NA,times=ncol(PPIWMdiffendlwithout)),
                             PPIWMdiffendlwithout)
rownames(PPIWMdiffendlheader)[1] <- "Author"

#Generation of a label matrix including group, n, BI baseline, BI endline,
#difference-in-differences and difference-of-endlines.

lab_mtrx_WMPPI <- cbind(rownames(PPIWMdiffdiffheader),
                       append("group",
                              round(PPIWMdiffdiffheader[,"group"],0)[2:10]),
                       append("n",
                              round(PPIWMdiffdiffheader[,"n"],0)[2:10]),
                       append("PPI (b)",
                              round(PPIWMdiffdiffheader[,"PPI (b)"],2)[2:10]),
```

```
                        append("PPI (e)",
                               round(PPIWMdiffdiffheader[,"PPI (e)"],2)[2:10]),
                        append("DID",
                               round(PPIWMdiffdiffheader[,"coef"],2)[2:10]),
                        append("DOE",
                               round(PPIWMdiffendlheader[,"coef"],2)[2:10]))

#Generation of an excel table which was manually processed and red in R again

write.xlsx(lab_mtrx_WMPPI, "C:/Users/Claudia/Desktop/R_Plot/lab_mtrx_WMPPI.xlsx")
lab_mtrx_WMPPI <- read_excel("C:/Users/Claudia/Desktop/R_Plot/lab_mtrx_WMPPIv2.xlsx")

#Generation of the forest plot with label matrix

forestplot2(mean=cbind(PPIWMdiffdiffheader[,"coef"], PPIWMdiffendlheader[,"coef"]),
            lower=cbind(PPIWMdiffdiffheader[,"lower"], PPIWMdiffendlheader[,"lower"]),
            upper=cbind(PPIWMdiffdiffheader[,"upper"], PPIWMdiffendlheader[,"upper"]),
            labeltext=lab_mtrx_WMPPI,
            txt_gp = fpTxtGp(label = gpar(cex = 0.7),
                             legend = gpar(cex = 0.7),
                             ticks = gpar(cex = 0.7)),
            is.summary=c(TRUE, rep(FALSE, times=nrow(PPIWMdiffdiffheader)-1)),
            clip=c(-1.5, 0.5),
            xticks=c(-1.5, -1, -0.5, 0, 0.5),
            boxsize=
              c(0.0,
                0.48,
                0,
                0.23,
                0,
                0.21,
                0,
                0.48,
                0,
                1),
            col=fpColors(box=c("darkred","darkblue")),
            fn.ci_norm=
              list(fpDrawNormalCI,
                   fpDrawNormalCI,
                   fpDrawNormalCI,
                   fpDrawNormalCI,
                   fpDrawNormalCI,
                   fpDrawNormalCI,
                   fpDrawNormalCI,
                   fpDrawNormalCI,
                   fpDrawNormalCI,
                   fpDrawDiamondCI),
            pch=13,
            new_page=TRUE,
            hrzl_lines = list("2" = gpar(col="#444444"),
                              "4" = gpar(col="#444444"),
                              "6" = gpar(col="#444444"),
                              "8" = gpar(col="#444444"),
                              "10" = gpar(col="#444444")),
            colgap = unit(5, "mm"),
            lineheight = unit(0.6, "cm"),
            line.margin = unit(0.2, "cm"),
            graphwidth = unit(10, "cm"),
            legend=c("difference-in-differences (DID)","difference-of-endlines (DOE)"),
            legend_args = fpLegend(pos = list(x=0.0, y=-0.25))
)

#Waste management with direct garbage collection - Breteau Index

#Generation of matrices including study group, n, BI baseline, BI endline
#and the coefficients and intervals of difference-in-differences and difference-of-endlines.

BIWMdiffdiffwith <-
  structure(
    c(1, 0, 1, 0, 1, 0, 1, 0, 1, 0, 1, 0, NA,
      NA, NA, 1689, 1580, 803, 790, 1000, 1000, 389, 390, 441, 448, NA,
      20, 37.5, 1.5991, 1.0278, 11.75, 9.75, 30.8, 22.2, 8, 11, 81.86, 78.79, NA,
      10.8, 46.9, 2.4646, 4.3158, 3.13, 6.25, 4.3, 21.4, 11, 24, 24.46, 21.49, NA,
      -18.6, NA, -2.4225, NA, -5.12, NA, -25.7, NA, -10, NA, -0.1, NA, -8.834,
      -18.6, NA, -2.4225, NA, -5.12, NA, -25.7, NA, -10, NA, -0.1, NA, -8.834,
      -18.6, NA, -2.4225, NA, -5.12, NA, -25.7, NA, -10, NA, -0.1, NA, -8.834),
    .Dim = c(13L, 7L),
    .Dimnames = list(c("Overgaard", "2016",
```

```
                     "Caprara", "2015",
                     "Abeyewickreme", "2012",
                     "Arunachalam", "2012",
                     "Castro", "2012",
                     "Kittayapong", "2012",
                     "Pooled"),
                   c("group", "n", "BI (b)", "BI (e)", "coef", "lower", "upper"))
  )

BIWMdiffendlwith <-
  structure(
    c(1, 0, 1, 0, 1, 0, 1, 0, 1, 0, 1, 0, NA,
      NA, NA, 1689, 1580, 803, 790, 1000, 1000, 389, 390, 441, 448, NA,
      20, 37.5, 1.5991, 1.0278, 11.75, 9.75, 30.8, 22.2, 8, 11, 81.86, 78.79, NA,
      10.8, 46.9, 2.4646, 4.3158, 3.13, 6.25, 4.3, 21.4, 11, 24, 24.46, 21.49, NA,
      -36.1, NA, -1.8512, NA, -3.12, NA, -17.1, NA, -13, NA, 2.97, NA, -6.179,
      -36.1, NA, -1.8512, NA, -3.12, NA, -17.1, NA, -13, NA, 2.97, NA, -6.179,
      -36.1, NA, -1.8512, NA, -3.12, NA, -17.1, NA, -13, NA, 2.97, NA, -6.179),
    .Dim = c(13L, 7L),
    .Dimnames = list(c("Overgaard", "2016",
                     "Caprara", "2015",
                     "Abeyewickreme", "2012",
                     "Arunachalam", "2012",
                     "Castro", "2012",
                     "Kittayapong", "2012",
                     "Pooled"),
                   c("group", "n", "BI (b)", "BI (e)", "coef", "lower", "upper"))
  )

#Introduction of a header for the columns

BIWMdiffdiffwithheader <- rbind(rep(NA,times=ncol(BIWMdiffdiffwith)),
                          BIWMdiffdiffwith)
rownames(BIWMdiffdiffwithheader)[1] <- "Author"
BIWMdiffendlwithheader <- rbind(rep(NA,times=ncol(BIWMdiffendlwith)),
                          BIWMdiffendlwith)
rownames(BIWMdiffendlwithheader)[1] <- "Author"

#Generation of a label matrix including group, n, BI baseline, BI endline,
#difference-in-differences and difference-of-endlines.

lab_mtrx_WMBIWith <- cbind(rownames(BIWMdiffdiffwithheader),
                     append("group",
                            round(BIWMdiffdiffwithheader[,"group"],0)[2:14]),
                     append("n",
                            round(BIWMdiffdiffwithheader[,"n"],0)[2:14]),
                     append("BI (b)",
                            round(BIWMdiffdiffwithheader[,"BI (b)"],1)[2:14]),
                     append("BI (e)",
                            round(BIWMdiffdiffwithheader[,"BI (e)"],1)[2:14]),
                     append("DID",
                            round(BIWMdiffdiffwithheader[,"coef"],1)[2:14]),
                     append("DOE",
                            round(BIWMdiffendlwithheader[,"coef"],1)[2:14]))

#Generation of an excel table which was manually processed and red in R again

write.xlsx(lab_mtrx_WMBIWith, "C:/Users/Claudia/Desktop/R_Plot/lab_mtrx_WMBIWith.xlsx")
lab_mtrx_WMBIWith <- read_excel("C:/Users/Claudia/Desktop/R_Plot/lab_mtrx_WMBIWithv2.xlsx")

#Generation of the forest plot with label matrix

forestplot2(mean=cbind(BIWMdiffdiffwithheader[,"coef"], BIWMdiffendlwithheader[,"coef"]),
            lower=cbind(BIWMdiffdiffwithheader[,"lower"], BIWMdiffendlwithheader[,"lower"]),
            upper=cbind(BIWMdiffdiffwithheader[,"upper"], BIWMdiffendlwithheader[,"upper"]),
            labeltext=lab_mtrx_WMBIWith,
            txt_gp = fpTxtGp(label = gpar(cex = 0.7),
                             legend = gpar(cex = 0.7),
                             ticks = gpar(cex = 0.7)),
            is.summary=c(TRUE, rep(FALSE, times=nrow(BIWMdiffdiffwithheader)-1)),
            clip=c(-40, 10),
            xticks=c(-40, -30, -20, -10, 0, 10),
            boxsize=
              c(0.0,
                0.6,
                0,
                0.43,
                0,
```

```
                0.31,
                0,
                0.34,
                0,
                0.25,
                0,
                0.26,
                0,
                1),
            col=fpColors(box=c("darkred","darkblue")),
            fn.ci_norm=
              list(fpDrawCircleCI,
                   fpDrawCircleCI,
                   fpDrawNormalCI,
                   fpDrawNormalCI,
                   fpDrawNormalCI,
                   fpDrawNormalCI,
                   fpDrawNormalCI,
                   fpDrawNormalCI,
                   fpDrawNormalCI,
                   fpDrawNormalCI,
                   fpDrawNormalCI,
                   fpDrawNormalCI,
                   fpDrawNormalCI,
                   fpDrawDiamondCI),
            pch=13,
            new_page=TRUE,
            hrzl_lines = list("2" = gpar(col="#444444"),
                              "4" = gpar(lty=2),
                              "6" = gpar(col="#444444"),
                              "8" = gpar(col="#444444"),
                              "10" = gpar(col="#444444"),
                              "12" = gpar(col="#444444"),
                              "14" = gpar(col="#444444")),
            colgap = unit(5, "mm"),
            lineheight = unit(0.6, "cm"),
            line.margin = unit(0.2, "cm"),
            graphwidth = unit(10, "cm"),
            legend=c("difference-in-differences (DID)","difference-of-endlines (DOE)"),
            legend_args = fpLegend(pos = list(x=0.0, y=-0.2))
)

#Waste management with direct garbage collection - Pupae Per Person Index

#Generation of matrices including study group, n, BI baseline, BI endline
#and the coefficients and intervals of difference-in-differences and difference-of-endlines.

PPIWMdiffdiffwith <-
  structure(
    c(1, 0, 1, 0, 1, 0, 1, 0, 1, 0, NA,
      NA, NA, 1689, 1580, 1000, 1000, 441, 448, 592, 600, NA,
      0, 0.06, 0.0229, 0.0104, 1.075, 0.729, 0.44, 0.42, 0.34, 0.33, NA,
      0.04, 0.36, 0.0292, 0.0539, 0.004, 0.355, 0.04, 0.4, 0.23, 0.15, NA,
      -0.26, NA, -0.0372, NA, -0.697, NA, -0.38, NA, 0.07, NA, -0.2408,
      -0.26, NA, -0.0372, NA, -0.697, NA, -0.38, NA, 0.07, NA, -0.2408,
      -0.26, NA, -0.0372, NA, -0.697, NA, -0.38, NA, 0.07, NA, -0.2408),
    .Dim = c(11L, 7L),
    .Dimnames = list(c("Overgaard", "2016",
                       "Caprara", "2015",
                       "Arunachalam", "2012",
                       "Kittayapong", "2012",
                       "Wai", "2012",
                       "Pooled"),
                     c("group", "n", "PPI (b)", "PPI (e)", "coef", "lower", "upper"))
  )

PPIWMdiffendlwith <-
  structure(
    c(1, 0, 1, 0, 1, 0, 1, 0, 1, 0, NA,
      NA, NA, 1689, 1580, 1000, 1000, 441, 448, 592, 600, NA,
      0, 0.06, 0.0229, 0.0104, 1.075, 0.729, 0.44, 0.42, 0.34, 0.33, NA,
      0.04, 0.36, 0.0292, 0.0539, 0.004, 0.355, 0.04, 0.4, 0.23, 0.15, NA,
      -0.32, NA, -0.0247, NA, -0.351, NA, -0.36, NA, 0.08, NA, -0.1371,
      -0.32, NA, -0.0247, NA, -0.351, NA, -0.36, NA, 0.08, NA, -0.1371,
      -0.32, NA, -0.0247, NA, -0.351, NA, -0.36, NA, 0.08, NA, -0.1371),
    .Dim = c(11L, 7L),
    .Dimnames = list(c("Overgaard", "2016",
                       "Caprara", "2015",
```

```
                      "Arunachalam", "2012",
                      "Kittayapong", "2012",
                      "Wai", "2012",
                      "Pooled"),
                    c("group", "n", "PPI (b)", "PPI (e)", "coef", "lower", "upper"))
  )

#Introduction of a header for the columns

PPIWMdiffdiffwithheader <- rbind(rep(NA,times=ncol(PPIWMdiffdiffwith)),
                                PPIWMdiffdiffwith)
rownames(PPIWMdiffdiffwithheader)[1] <- "Author"
PPIWMdiffendlwithheader <- rbind(rep(NA,times=ncol(PPIWMdiffendlwith)),
                                PPIWMdiffendlwith)
rownames(PPIWMdiffendlwithheader)[1] <- "Author"

#Generation of a label matrix including group, n, BI baseline, BI endline,
#difference-in-differences and difference-of-endlines.

lab_mtrx_WMPPIWith <- cbind(rownames(PPIWMdiffdiffwithheader),
                           append("group",
                                  round(PPIWMdiffdiffwithheader[,"group"],0)[2:12]),
                           append("n",
                                  round(PPIWMdiffdiffwithheader[,"n"],0)[2:12]),
                           append("PPI (b)",
                                  round(PPIWMdiffdiffwithheader[,"PPI (b)"],2)[2:12]),
                           append("PPI (e)",
                                  round(PPIWMdiffdiffwithheader[,"PPI (e)"],2)[2:12]),
                           append("DID",
                                  round(PPIWMdiffdiffwithheader[,"coef"],2)[2:12]),
                           append("DOE",
                                  round(PPIWMdiffendlwithheader[,"coef"],2)[2:12]))

#Generation of an excel table which was manually processed and red in R again

write.xlsx(lab_mtrx_WMPPIWith, "C:/Users/Claudia/Desktop/R_Plot/lab_mtrx_WMPPIWith.xlsx")
lab_mtrx_WMPPIWith <- read_excel("C:/Users/Claudia/Desktop/R_Plot/lab_mtrx_WMPPIWithv2.xlsx")

#Generation of the forest plot with label matrix

forestplot2(mean=cbind(PPIWMdiffdiffwithheader[,"coef"], PPIWMdiffendlwithheader[,"coef"]),
            lower=cbind(PPIWMdiffdiffwithheader[,"lower"], PPIWMdiffendlwithheader[,"lower"]),
            upper=cbind(PPIWMdiffdiffwithheader[,"upper"], PPIWMdiffendlwithheader[,"upper"]),
            labeltext=lab_mtrx_WMPPIWith,
            txt_gp = fpTxtGp(label = gpar(cex = 0.7),
                             legend = gpar(cex = 0.7),
                             ticks = gpar(cex = 0.7)),
            is.summary=c(TRUE, rep(FALSE, times=nrow(PPIWMdiffdiffwithheader)-1)),
            clip=c(-0.8, 0.2),
            xticks=c(-0.8, -0.6, -0.4, -0.2, 0, 0.2),
            boxsize=
              c(0.0,
                0.6,
                0,
                0.47,
                0,
                0.36,
                0,
                0.27,
                0,
                0.30,
                0,
                1),
            col=fpColors(box=c("darkred","darkblue")),
            fn.ci_norm=
              list(fpDrawCircleCI,
                   fpDrawCircleCI,
                   fpDrawNormalCI,
                   fpDrawNormalCI,
                   fpDrawNormalCI,
                   fpDrawNormalCI,
                   fpDrawNormalCI,
                   fpDrawNormalCI,
                   fpDrawNormalCI,
                   fpDrawNormalCI,
                   fpDrawNormalCI,
                   fpDrawDiamondCI),
            pch=13,
```

```
            new_page=TRUE,
            hrzl_lines = list("2" = gpar(col="#444444"),
                              "4" = gpar(lty=2),
                              "6" = gpar(col="#444444"),
                              "8" = gpar(col="#444444"),
                              "10" = gpar(col="#444444"),
                              "12" = gpar(col="#444444")),
            colgap = unit(5, "mm"),
            lineheight = unit(0.6, "cm"),
            line.margin = unit(0.2, "cm"),
            graphwidth = unit(10, "cm"),
            legend=c("difference-in-differences (DID)","difference-of-endlines (DOE)"),
            legend_args = fpLegend(pos = list(x=0.0, y=-0.2))
)

#Elimination of breeding places - Breteau Index

#Generation of matrices including study group, n, BI baseline, BI endline
#and the coefficients and intervals of difference-in-differences and difference-of-endlines.

BIELdiffdiffwithout <-
  structure(
    c(1, 0, 1, 0, 1, 0, 1, 0, NA,
      1000, 1000, 441, 448, 500, 500, 8422, 10748, NA,
      3.4, 2.64, 81.86, 78.79, 17.1, 10.9, 0.27, 0.2, NA,
      12.02, 13.77, 24.46, 21.49, 17.8, 12.7, 0.28, 0.52, NA,
      -2.51, NA, -0.1, NA, -1.1, NA, -0.31, NA, -0.527,
      -2.51, NA, -0.1, NA, -1.1, NA, -0.31, NA, -0.527,
      -2.51, NA, -0.1, NA, -1.1, NA, -0.31, NA, -0.527),
    .Dim = c(9L, 7L),
    .Dimnames = list(c("Basso", "2015",
                       "Kittayapong", "2012",
                       "Tun-Lin (Peru)", "2009",
                       "Vanlerberghe", "2009",
                       "Pooled"),
                     c("group", "n", "BI (b)", "BI (e)", "coef", "lower", "upper"))
  )

BIELdiffendlwithout <-
  structure(
    c(1, 0, 1, 0, 1, 0, 1, 0, NA,
      1000, 1000, 441, 448, 500, 500, 8422, 10748, NA,
      3.4, 2.64, 81.86, 78.79, 17.1, 10.9, 0.27, 0.2, NA,
      12.02, 13.77, 24.46, 21.49, 17.8, 12.7, 0.28, 0.52, NA,
      -1.75, NA, 2.97, NA, 5.1, NA, -0.24, NA, -0.015,
      -1.75, NA, 2.97, NA, 5.1, NA, -0.24, NA, -0.015,
      -1.75, NA, 2.97, NA, 5.1, NA, -0.24, NA, -0.015),
    .Dim = c(9L, 7L),
    .Dimnames = list(c("Basso", "2015",
                       "Kittayapong", "2012",
                       "Tun-Lin (Peru)", "2009",
                       "Vanlerberghe", "2009",
                       "Pooled"),
                     c("group", "n", "BI (b)", "BI (e)", "coef", "lower", "upper"))
  )

#Introduction of a header for the columns

BIELdiffdiffheader <- rbind(rep(NA,times=ncol(BIELdiffdiffwithout)),
                            BIELdiffdiffwithout)
rownames(BIELdiffdiffheader)[1] <- "Author"
BIELdiffendlheader <- rbind(rep(NA,times=ncol(BIELdiffendlwithout)),
                            BIELdiffendlwithout)
rownames(BIELdiffendlheader)[1] <- "Author"

#Generation of a label matrix including group, n, BI baseline, BI endline,
#difference-in-differences and difference-of-endlines.

lab_mtrx_ELBI <- cbind(rownames(BIELdiffdiffheader),
                       append("group",
                              round(BIELdiffdiffheader[,"group"],0)[2:10]),
                       append("n",
                              round(BIELdiffdiffheader[,"n"],0)[2:10]),
                       append("BI (b)",
                              round(BIELdiffdiffheader[,"BI (b)"],1)[2:10]),
                       append("BI (e)",
                              round(BIELdiffdiffheader[,"BI (e)"],1)[2:10]),
                       append("DID",
```

```
                              round(BIELdiffdiffheader[,"coef"],1)[2:10]),
                       append("DOE",
                              round(BIELdiffendlheader[,"coef"],1)[2:10]))

#Generation of an excel table which was manually processed and red in R again

write.xlsx(lab_mtrx_ELBI, "C:/Users/Claudia/Desktop/R_Plot/lab_mtrx_ELBI.xlsx")
lab_mtrx_ELBI <- read_excel("C:/Users/Claudia/Desktop/R_Plot/lab_mtrx_ELBIv2.xlsx")

#Generation of the forest plot with label matrix

forestplot2(mean=cbind(BIELdiffdiffheader[,"coef"], BIELdiffendlheader[,"coef"]),
            lower=cbind(BIELdiffdiffheader[,"lower"], BIELdiffendlheader[,"lower"]),
            upper=cbind(BIELdiffdiffheader[,"upper"], BIELdiffendlheader[,"upper"]),
            labeltext=lab_mtrx_ELBI,
            txt_gp = fpTxtGp(label = gpar(cex = 0.7),
                             legend = gpar(cex = 0.7),
                             ticks = gpar(cex = 0.7)),
            is.summary=c(TRUE, rep(FALSE, times=nrow(BIELdiffdiffheader)-1)),
            clip=c(-5, 7),
            xticks=c(-5, -3, -1, 1, 3, 5, 7),
            boxsize=
              c(0.0,
                0.25,
                0,
                0.22,
                0,
                0.23,
                0,
                0.70,
                0,
                1),
            col=fpColors(box=c("darkred","darkblue")),
            fn.ci_norm=
              list(fpDrawNormalCI,
                   fpDrawNormalCI,
                   fpDrawNormalCI,
                   fpDrawNormalCI,
                   fpDrawNormalCI,
                   fpDrawNormalCI,
                   fpDrawNormalCI,
                   fpDrawNormalCI,
                   fpDrawNormalCI,
                   fpDrawDiamondCI),
            pch=13,
            new_page=TRUE,
            hrzl_lines = list("2" = gpar(col="#444444"),
                              "4" = gpar(col="#444444"),
                              "6" = gpar(col="#444444"),
                              "8" = gpar(col="#444444"),
                              "10" = gpar(col="#444444")),
            colgap = unit(5, "mm"),
            lineheight = unit(0.6, "cm"),
            line.margin = unit(0.2, "cm"),
            graphwidth = unit(10, "cm"),
            legend=c("difference-in-differences (DID)","difference-of-endlines (DOE)"),
            legend_args = fpLegend(pos = list(x=0.0, y=-0.25))
)

#Elimination of breeding places - Pupae Per Person Index

#Generation of matrices including study group, n, BI baseline, BI endline
#and the coefficients and intervals of difference-in-differences and difference-of-endlines.

PPIELdiffdiffwithout <-
  structure(
    c(1, 0, 1, 0, 1, 0, 1, 0, NA,
      2000, 2000, 441, 448, 500, 500, 8422, 10748, NA,
      0.048, 0.016, 0.44, 0.42, 0.11, 0.21, 0.44, 0.29, NA,
      0.13, 0.14, 0.04, 0.4, 0.37, 0.11, 0.36, 1.4, NA,
      -0.042, NA, -0.38, NA, 0.36, NA, -1.19, NA, -0.952,
      -0.042, NA, -0.38, NA, 0.36, NA, -1.19, NA, -0.952,
      -0.042, NA, -0.38, NA, 0.36, NA, -1.19, NA, -0.952),
    .Dim = c(9L, 7L),
    .Dimnames = list(c("Basso", "2015",
                       "Kittayapong", "2012",
                       "Tun-Lin (Peru)", "2009",
                       "Vanlerberghe", "2009",
```

```
                          "Pooled"),
                        c("group", "n", "PPI (b)", "PPI (e)", "coef", "lower", "upper"))
  )

PPIELdiffendlwithout <-
  structure(
    c(1, 0, 1, 0, 1, 0, 1, 0, NA,
      2000, 2000, 441, 448, 500, 500, 8422, 10748, NA,
      0.048, 0.016, 0.44, 0.42, 0.11, 0.21, 0.44, 0.29, NA,
      0.13, 0.14, 0.04, 0.4, 0.37, 0.11, 0.36, 1.4, NA,
      -0.01, NA, -0.36, NA, 0.26, NA, -1.04, NA, -0.832,
      -0.01, NA, -0.36, NA, 0.26, NA, -1.04, NA, -0.832,
      -0.01, NA, -0.36, NA, 0.26, NA, -1.04, NA, -0.832),
    .Dim = c(9L, 7L),
    .Dimnames = list(c("Basso", "2015",
                          "Kittayapong", "2012",
                          "Tun-Lin (Peru)", "2009",
                          "Vanlerberghe", "2009",
                          "Pooled"),
                        c("group", "n", "PPI (b)", "PPI (e)", "coef", "lower", "upper"))
  )

#Introduction of a header for the columns

PPIELdiffdiffheader <- rbind(rep(NA,times=ncol(PPIELdiffdiffwithout)),
                             PPIELdiffdiffwithout)
rownames(PPIELdiffdiffheader)[1] <- "Author"
PPIELdiffendlheader <- rbind(rep(NA,times=ncol(PPIELdiffendlwithout)),
                             PPIELdiffendlwithout)
rownames(PPIELdiffendlheader)[1] <- "Author"

#Generation of a label matrix including group, n, BI baseline, BI endline,
#difference-in-differences and difference-of-endlines.

lab_mtrx_ELPPI <- cbind(rownames(PPIELdiffdiffheader),
                        append("group",
                               round(PPIELdiffdiffheader[,"group"],0)[2:10]),
                        append("n",
                               round(PPIELdiffdiffheader[,"n"],0)[2:10]),
                        append("PPI (b)",
                               round(PPIELdiffdiffheader[,"PPI (b)"],2)[2:10]),
                        append("PPI (e)",
                               round(PPIELdiffdiffheader[,"PPI (e)"],2)[2:10]),
                        append("DID",
                               round(PPIELdiffdiffheader[,"coef"],2)[2:10]),
                        append("DOE",
                               round(PPIELdiffendlheader[,"coef"],2)[2:10]))

#Generation of an excel table which was manually processed and red in R again

write.xlsx(lab_mtrx_ELPPI, "C:/Users/Claudia/Desktop/R_Plot/lab_mtrx_ELPPI.xlsx")
lab_mtrx_ELPPI <- read_excel("C:/Users/Claudia/Desktop/R_Plot/lab_mtrx_ELPPIv2.xlsx")

#Generation of the forest plot with label matrix

forestplot2(mean=cbind(PPIELdiffdiffheader[,"coef"], PPIELdiffendlheader[,"coef"]),
            lower=cbind(PPIELdiffdiffheader[,"lower"], PPIELdiffendlheader[,"lower"]),
            upper=cbind(PPIELdiffdiffheader[,"upper"], PPIELdiffendlheader[,"upper"]),
            labeltext=lab_mtrx_ELPPI,
            txt_gp = fpTxtGp(label = gpar(cex = 0.7),
                             legend = gpar(cex = 0.7),
                             ticks = gpar(cex = 0.7)),
            is.summary=c(TRUE, rep(FALSE, times=nrow(PPIELdiffdiffheader)-1)),
            clip=c(-1.5, 0.5),
            xticks=c(-1.5, -1, -0.5, 0, 0.5),
            boxsize=
              c(0.0,
                0.27,
                0,
                0.22,
                0,
                0.22,
                0,
                0.68,
                0,
                1),
            col=fpColors(box=c("darkred","darkblue")),
            fn.ci_norm=
```

```
              list(fpDrawNormalCI,
                   fpDrawNormalCI,
                   fpDrawNormalCI,
                   fpDrawNormalCI,
                   fpDrawNormalCI,
                   fpDrawNormalCI,
                   fpDrawNormalCI,
                   fpDrawNormalCI,
                   fpDrawNormalCI,
                   fpDrawDiamondCI),
            pch=13,
            new_page=TRUE,
            hrzl_lines = list("2" = gpar(col="#444444"),
                              "4" = gpar(col="#444444"),
                              "6" = gpar(col="#444444"),
                              "8" = gpar(col="#444444"),
                              "10" = gpar(col="#444444")),
            colgap = unit(5, "mm"),
            lineheight = unit(0.6, "cm"),
            line.margin = unit(0.2, "cm"),
            graphwidth = unit(10, "cm"),
            legend=c("difference-in-differences (DID)","difference-of-endlines (DOE)"),
            legend_args = fpLegend(pos = list(x=0.0, y=-0.25))
)

#Pooled estimates - Breteau Index

BI_pooled_overview_DID <-
  structure(
    c(-7.897530338, -3.616872038, -8.834022567, -0.448497092, -0.52697862,
      -7.897530338, -3.616872038, -8.834022567, -0.448497092, -0.52697862,
      -7.897530338, -3.616872038, -8.834022567, -0.448497092, -0.52697862),
    .Dim = c(5L, 3L),
    .Dimnames = list(c("container covers without insecticides",
                       "container covers with insecticides",
                       "waste management with direct garbage collection",
                       "waste management without direct garbage collection",
                       "elimination of breeding places"),
                     c("coef", "lower", "upper"))
  )

BI_pooled_overview_DOE <-
  structure(
    c(-5.039377642, 0.636872038, -6.179179695, -5.378552679, -0.015632508,
      -5.039377642, 0.636872038, -6.179179695, -5.378552679, -0.015632508,
      -5.039377642, 0.636872038, -6.179179695, -5.378552679, -0.015632508),
    .Dim = c(5L, 3L),
    .Dimnames = list(c("container covers without insecticides",
                       "container covers with insecticides",
                       "waste management with direct garbage collection",
                       "waste management without direct garbage collection",
                       "elimination of breeding places"),
                     c("coef", "lower", "upper"))
  )

#Introduction of a header for the columns

BI_pooled_DID_header <- rbind(rep(NA,times=ncol(BI_pooled_overview_DID)),
                             BI_pooled_overview_DID)
BI_pooled_DOE_header <- rbind(rep(NA,times=ncol(BI_pooled_overview_DOE)),
                                 BI_pooled_overview_DOE)

#Generation of a label matrix including group, n, BI baseline, BI endline,
#difference-in-differences and difference-of-endlines.

lab_mtrx_pooledBI <- cbind(rownames(BI_pooled_DID_header),
                       append("DID",
                              round(BI_pooled_DID_header[,"coef"],1)[2:6]),
                       append("DOE",
                              round(BI_pooled_DOE_header[,"coef"],1)[2:6]))

#Generation of the forest plot with label matrix

forestplot2(mean=cbind(BI_pooled_DID_header[,"coef"], BI_pooled_DOE_header[,"coef"]),
            lower=cbind(BI_pooled_DID_header[,"lower"], BI_pooled_DOE_header[,"lower"]),
            upper=cbind(BI_pooled_DID_header[,"upper"], BI_pooled_DOE_header[,"upper"]),
            labeltext=lab_mtrx_pooledBI,
            txt_gp = fpTxtGp(label = gpar(cex = 0.7),
```

```
                                legend = gpar(cex = 0.7),
                                ticks = gpar(cex = 0.7)),
            is.summary=c(TRUE, rep(FALSE, times=nrow(BI_pooled_DID_header)-1)),
            clip=c(-10, 2),
            xticks=c(-10, -8, -6, -4, -2, 0, 2),
            boxsize=
              c(0.9,
                0.9,
                0.9,
                0.9,
                0.9,
                0.9),
            col=fpColors(box=c("darkred","darkblue")),
            fn.ci_norm=
              list(fpDrawDiamondCI,
                   fpDrawDiamondCI,
                   fpDrawDiamondCI,
                   fpDrawDiamondCI,
                   fpDrawDiamondCI,
                   fpDrawDiamondCI),
            pch=13,
            new_page=TRUE,
            hrzl_lines = list("2" = gpar(col="#444444"),
                              "3" = gpar(col="#444444"),
                              "4" = gpar(col="#444444"),
                              "5" = gpar(col="#444444"),
                              "6" = gpar(col="#444444")),
            colgap = unit(5, "mm"),
            lineheight = unit(0.8, "cm"),
            line.margin = unit(0.4, "cm"),
            graphwidth = unit(10, "cm"),
            legend=c("difference-in-differences (DID)","difference-of-endlines (DOE)"),
            legend_args = fpLegend(pos = list(x=0.0, y=-0.3))
)

#Pooled estimates - Pupae Per Person Index

PPI_pooled_overview_DID <-
  structure(
    c(-0.18318713, -0.83268121, -0.240814531, -0.563559196, -0.952496779,
      -0.18318713, -0.83268121, -0.240814531, -0.563559196, -0.952496779,
      -0.18318713, -0.83268121, -0.240814531, -0.563559196, -0.952496779),
    .Dim = c(5L, 3L),
    .Dimnames = list(c("container covers without insecticides",
                       "container covers with insecticides",
                       "waste management with direct garbage collection",
                       "waste management without direct garbage collection",
                       "elimination of breeding places"),
                     c("coef", "lower", "upper"))
  )

PPI_pooled_overview_DOE <-
  structure(
    c(-0.100234232, 0.088522167, -0.137064531, -0.545940259, -0.832405337,
      -0.100234232, 0.088522167, -0.137064531, -0.545940259, -0.832405337,
      -0.100234232, 0.088522167, -0.137064531, -0.545940259, -0.832405337),
    .Dim = c(5L, 3L),
    .Dimnames = list(c("container covers without insecticides",
                       "container covers with insecticides",
                       "waste management with direct garbage collection",
                       "waste management without direct garbage collection",
                       "elimination of breeding places"),
                     c("coef", "lower", "upper"))
  )

#Introduction of a header for the columns

PPI_pooled_DID_header <- rbind(rep(NA,times=ncol(PPI_pooled_overview_DID)),
                               PPI_pooled_overview_DID)
PPI_pooled_DOE_header <- rbind(rep(NA,times=ncol(PPI_pooled_overview_DOE)),
                                PPI_pooled_overview_DOE)

#Generation of a label matrix including group, n, BI baseline, BI endline,
#difference-in-differences and difference-of-endlines.

lab_mtrx_pooledPPI <- cbind(rownames(PPI_pooled_DID_header),
                            append("DID",
                                   round(PPI_pooled_DID_header[,"coef"],2)[2:6]),
```

```
                           append("DOE",
                                  round(PPI_pooled_DOE_header[,"coef"],2)[2:6]))
```

#Generation of the forest plot with label matrix

```
forestplot2(mean=cbind(PPI_pooled_DID_header[,"coef"], PPI_pooled_DOE_header[,"coef"]),
            lower=cbind(PPI_pooled_DID_header[,"lower"], PPI_pooled_DOE_header[,"lower"]),
            upper=cbind(PPI_pooled_DID_header[,"upper"], PPI_pooled_DOE_header[,"upper"]),
            labeltext=lab_mtrx_pooledPPI,
            txt_gp = fpTxtGp(label = gpar(cex = 0.7),
                             legend = gpar(cex = 0.7),
                             ticks = gpar(cex = 0.7)),
            is.summary=c(TRUE, rep(FALSE, times=nrow(PPI_pooled_DID_header)-1)),
            clip=c(-1, 0.2),
            xticks=c(-1, -0.8, -0.6, -0.4, -0.2, 0, 0.2),
            boxsize=
              c(0.9,
                0.9,
                0.9,
                0.9,
                0.9,
                0.9),
            col=fpColors(box=c("darkred","darkblue")),
            fn.ci_norm=
              list(fpDrawDiamondCI,
                   fpDrawDiamondCI,
                   fpDrawDiamondCI,
                   fpDrawDiamondCI,
                   fpDrawDiamondCI,
                   fpDrawDiamondCI),
            pch=13,
            new_page=TRUE,
            hrzl_lines = list("2" = gpar(col="#444444"),
                              "3" = gpar(col="#444444"),
                              "4" = gpar(col="#444444"),
                              "5" = gpar(col="#444444"),
                              "6" = gpar(col="#444444")),
            colgap = unit(5, "mm"),
            lineheight = unit(0.8, "cm"),
            line.margin = unit(0.4, "cm"),
            graphwidth = unit(10, "cm"),
            legend=c("difference-in-differences (DID)","difference-of-endlines (DOE)"),
            legend_args = fpLegend(pos = list(x=0.0, y=-0.3))
)
```
