# Supplementary material for: Environmental methods for dengue vector control – A systematic review and meta-analysis
Source: PLoS Negl Trop Dis. 2019 Jul 11;13(7):e0007420. doi: 10.1371/journal.pntd.0007420 (PMC6650086; doi:10.1371/journal.pntd.0007420)
Supplement: S3 Appendix — (PDF) [file pntd.0007420.s003.pdf]

## S3 Appendix. Quality assessment

| Section/Topic | Item | Overgaard 2016 | | Andersson 2015 | | Basso 2015 | | Caprara 2015 | | Mitchell-Foster 2015 | |
|---|---|---|---|---|---|---|---|---|---|---|---|
| **Title and abstract** | | | | | | | | | | | |
| | **1a** | yes | | yes | | no | | yes | | no | |
| | **1b** | yes | good | yes | good | yes | ok | yes | ok | yes | good |
| **Introduction** | | | | | | | | | | | |
| **Background** | **2a** | yes | good | yes | good | yes | good | yes | good | yes | good |
| **Objectives** | **2b** | yes | good | yes | good | yes | good | yes | good | yes | good |
| **Methods** | | | | | | | | | | | |
| **Trial design** | **3a** | yes | good | yes | good | yes | good | yes | good | yes | good |
| | **3b** | NR | | NR | | NR | | NR | | NR | |
| **Participants** | **4a** | yes | good | yes | good | yes | good | no | | yes | ok |
| | **4b** | yes | good | yes | bad | yes | good | yes | good | yes | good |
| **Interventions** | **5** | yes | good | yes | good | yes | good | yes | good | yes | good |
| **Outcomes** | **6a** | yes | good | yes | good | yes | bad | no | | yes | good |
| | **6b** | NR | | NR | | NR | | NR | | NR | |
| **Sample size** | **7a** | yes | good | yes | good | yes | good | no | | yes | ok |
| | **7b** | NR | | NR | | NR | | NR | | NR | |
| **Randomisation** | | | | | | | | | | | |
| **Sequence generation** | **8a** | yes | good | yes | good | no | | yes | good | yes | good |
| | **8b** | yes | good | no | | yes | good | yes | ok | yes | good |
| **Allocation** | **9** | yes | ok | yes | good | yes | ok | yes | ok | yes | good |
| **Implementation** | **10** | yes | good | yes | good | no | | yes | bad | yes | ok |
| **Blinding** | **11a** | NR | | yes | good | NR | | NR | | NR | |
| | **11b** | NR | | NR | | NR | | NR | | NR | |
| **Statistical methods** | **12a** | yes | good | yes | good | yes | ok | yes | bad | yes | good |
| | **12b** | NR | | yes | good | NR | | NR | | NR | |
| **Results** | | | | | | | | | | | |
| **Participant flow** | **13a** | yes | good | yes | good | yes | bad | no | | yes | bad |
| | **13b** | yes | good | yes | bad | no | | no | | no | |
| **Recruitment** | **14a** | yes | good | yes | good | yes | good | yes | ok | yes | good |
| | **14b** | NR | | NR | | NR | | NR | | NR | |
| **Baseline data** | **15** | yes | good | yes | ok | no | | yes | ok | no | |
| **Numbers analysed** | **16** | yes | good | yes | good | yes | bad | yes | bad | yes | bad |
| **Outcomes & estimation** | **17a** | yes | good | yes | good | yes | ok | yes | bad | yes | good |
| | **17b** | NR | | yes | good | yes | good | no | | NR | |
| **Ancillary analyses** | **18** | NR | | yes | good | NR | | NR | | NR | |
| **Harms** | **19** | yes | good | no | | no | | no | | no | |
| **Discussion** | | | | | | | | | | | |
| **Limitations** | **20** | yes | good | yes | good | yes | good | yes | ok | yes | bad |
| **Generalisability** | **21** | yes | ok | yes | good | yes | ok | yes | ok | yes | ok |
| **Interpretation** | **22** | yes | good | yes | ok | yes | good | yes | ok | yes | good |
| **Other information** | | | | | | | | | | | |
| **Registration** | **23** | yes | | yes | | no | | no | | no | |
| **Protocol** | **24** | no | | no | | no | | no | | no | |
| **Funding** | **25** | yes | | yes | | yes | | yes | | yes | |

**S3 Appendix. Quality assessment of included studies using the CONSORT 2010 checklist as a framework.** NR: Not Relevant. (11)

| Section/Topic | Item | Quintero 2015 | | Abeyewick-reme 2012 | | Arunachalam 2012 | | Castro 2012 | | Kittayapong 2012 | |
|---|---|---|---|---|---|---|---|---|---|---|---|
| **Title and abstract** | | | | | | | | | | | |
| | **1a** | yes | | no | | no | | yes | | no | |
| | **1b** | yes | good | yes | good | yes | good | yes | ok | yes | ok |
| **Introduction** | | | | | | | | | | | |
| **Background** | **2a** | yes | ok | yes | good | yes | good | yes | ok | yes | ok |
| **Objectives** | **2b** | yes | good | yes | ok | yes | good | yes | ok | yes | ok |
| **Methods** | | | | | | | | | | | |
| **Trial design** | **3a** | yes | good | yes | good | yes | good | yes | good | yes | good |
| | **3b** | NR | | NR | | NR | | NR | | NR | |
| **Participants** | **4a** | no | | yes | good | yes | ok | no | | no | |
| | **4b** | yes | good | yes | ok | yes | good | yes | good | yes | good |
| **Interventions** | **5** | yes | good | yes | good | yes | good | yes | good | yes | good |
| **Outcomes** | **6a** | yes | good | yes | bad | yes | bad | yes | bad | yes | ok |
| | **6b** | NR | | NR | | NR | | NR | | NR | |
| **Sample size** | **7a** | yes | good | yes | ok | yes | good | yes | ok | yes | good |
| | **7b** | yes | good | NR | | NR | | NR | | NR | |
| **Randomisation** | | | | | | | | | | | |
| **Sequence generation** | **8a** | yes | good | yes | good | yes | good | no | | yes | bad |
| | **8b** | yes | good | yes | good | yes | good | yes | ok | yes | ok |
| **Allocation** | **9** | yes | good | yes | good | yes | good | no | | no | |
| **Implementation** | **10** | yes | bad | no | | yes | ok | no | | no | |
| **Blinding** | **11a** | NR | | NR | | NR | | NR | | NR | |
| | **11b** | NR | | NR | | NR | | NR | | NR | |
| **Statistical methods** | **12a** | yes | good | yes | ok | yes | ok | yes | good | yes | good |
| | **12b** | NR | | NR | | NR | | NR | | NR | |
| **Results** | | | | | | | | | | | |
| **Participant flow** | **13a** | yes | good | yes | bad | no | | yes | ok | yes | bad |
| | **13b** | yes | good | no | | no | | yes | good | no | |
| **Recruitment** | **14a** | yes | good | yes | good | yes | bad | yes | good | yes | good |
| | **14b** | NR | | NR | | NR | | NR | | NR | |
| **Baseline data** | **15** | no | | yes | good | no | | yes | good | yes | ok |
| **Numbers analysed** | **16** | yes | good | yes | bad | yes | bad | yes | good | yes | bad |
| **Outcomes & estimation** | **17a** | yes | good | yes | good | yes | good | yes | good | yes | bad |
| | **17b** | yes | good | yes | good | yes | ok | yes | ok | no | |
| **Ancillary analyses** | **18** | NR | | yes | ok | NR | | NR | | NR | |
| **Harms** | **19** | no | | no | | no | | no | | no | |
| **Discussion** | | | | | | | | | | | |
| **Limitations** | **20** | yes | good | yes | bad | no | | yes | bad | yes | bad |
| **Generalisability** | **21** | yes | good | yes | good | no | | yes | ok | yes | good |
| **Interpretation** | **22** | yes | good | yes | good | yes | ok | yes | good | yes | ok |
| **Other information** | | | | | | | | | | | |
| **Registration** | **23** | no | | no | | no | | no | | no | |
| **Protocol** | **24** | no | | no | | no | | no | | no | |
| **Funding** | **25** | yes | | no | | yes | | yes | | yes | |

**S3 Appendix. Quality assessment of included studies using the CONSORT 2010 checklist as a framework.** NR: Not Relevant. Continued.

| Section/Topic | Item | Rizzo 2012 | | Tana 2012 | | Wai 2012 | | Kusumawathie 2009 | | Tun-Lin 2009 | |
|---|---|---|---|---|---|---|---|---|---|---|---|
| **Title and abstract** | | | | | | | | | | | |
| | **1a** | no | | no | | no | | no | | yes | |
| | **1b** | yes | good | yes | ok | yes | good | no | | yes | good |
| **Introduction** | | | | | | | | | | | |
| **Background** | **2a** | yes | ok | yes | ok | yes | ok | yes | ok | yes | bad |
| **Objectives** | **2b** | yes | good | yes | good | yes | good | yes | good | yes | ok |
| **Methods** | | | | | | | | | | | |
| **Trial design** | **3a** | yes | good | yes | good | yes | good | yes | good | yes | good |
| | **3b** | NR | | NR | | NR | | NR | | NR | |
| **Participants** | **4a** | yes | ok | yes | ok | no | | NR | | no | |
| | **4b** | yes | good | yes | good | yes | good | yes | ok | yes | bad |
| **Interventions** | **5** | yes | good | yes | good | yes | ok | yes | good | yes | good |
| **Outcomes** | **6a** | yes | good | yes | ok | yes | ok | yes | bad | yes | bad |
| | **6b** | NR | | NR | | NR | | NR | | NR | |
| **Sample size** | **7a** | yes | good | no | | yes | ok | no | | yes | good |
| | **7b** | NR | | NR | | NR | | NR | | NR | |
| **Randomisation** | | | | | | | | | | | |
| **Sequence generation** | **8a** | yes | good | yes | good | no | | no | | no | |
| | **8b** | yes | good | yes | good | yes | ok | yes | ok | yes | ok |
| **Allocation** | **9** | yes | good | yes | good | no | | no | | yes | ok |
| **Implementation** | **10** | no | | no | | no | | yes | bad | no | |
| **Blinding** | **11a** | NR | | NR | | NR | | NR | | NR | |
| | **11b** | NR | | NR | | NR | | NR | | NR | |
| **Statistical methods** | **12a** | yes | ok | yes | ok | no | | yes | ok | yes | good |
| | **12b** | NR | | NR | | NR | | NR | | NR | |
| **Results** | | | | | | | | | | | |
| **Participant flow** | **13a** | yes | bad | no | | no | | yes | ok | yes | good |
| | **13b** | yes | ok | no | | no | | no | | yes | good |
| **Recruitment** | **14a** | yes | good | yes | good | yes | bad | yes | good | yes | good |
| | **14b** | NR | | NR | | NR | | NR | | NR | |
| **Baseline data** | **15** | yes | bad | no | | yes | bad | no | | no | |
| **Numbers analysed** | **16** | yes | ok | yes | bad | yes | bad | no | | yes | good |
| **Outcomes & estimation** | **17a** | yes | ok | yes | bad | yes | ok | yes | ok | yes | good |
| | **17b** | NR | | no | | yes | good | no | | NR | |
| **Ancillary analyses** | **18** | NR | | NR | | NR | | NR | | NR | |
| **Harms** | **19** | no | | no | | no | | no | | no | |
| **Discussion** | | | | | | | | | | | |
| **Limitations** | **20** | yes | good | no | | yes | bad | yes | ok | yes | ok |
| **Generalisability** | **21** | yes | bad | no | | yes | bad | yes | ok | yes | ok |
| **Interpretation** | **22** | yes | good | yes | bad | yes | good | yes | bad | yes | good |
| **Other information** | | | | | | | | | | | |
| **Registration** | **23** | no | | no | | no | | no | | no | |
| **Protocol** | **24** | no | | no | | no | | no | | no | |
| **Funding** | **25** | yes | | yes | | yes | | yes | | no | |

**S3 Appendix. Quality assessment of included studies using the CONSORT 2010 checklist as a framework.** NR: Not Relevant. Continued.

| Section/Topic | Item | Vanlerberghe 2009 | | Toledo 2007 | | Kroeger 2006 | | Leontsini 1993 | |
|---|---|---|---|---|---|---|---|---|---|
| **Title and abstract** | | | | | | | | | |
| | **1a** | yes | | no | | yes | | no | |
| | **1b** | yes | good | yes | bad | yes | good | yes | bad |
| **Introduction** | | | | | | | | | |
| **Background** | **2a** | yes | good | yes | good | yes | bad | yes | good |
| **Objectives** | **2b** | yes | good | yes | ok | yes | ok | yes | ok |
| **Methods** | | | | | | | | | |
| **Trial design** | **3a** | yes | good | yes | good | yes | good | yes | good |
| | **3b** | NR | | NR | | NR | | NR | |
| **Participants** | **4a** | yes | bad | yes | ok | yes | bad | no | |
| | **4b** | yes | good | yes | good | yes | good | yes | good |
| **Interventions** | **5** | yes | good | yes | good | yes | good | yes | good |
| **Outcomes** | **6a** | yes | good | yes | bad | yes | good | yes | ok |
| | **6b** | NR | | NR | | NR | | NR | |
| **Sample size** | **7a** | yes | ok | no | | yes | ok | no | |
| | **7b** | NR | | NR | | NR | | NR | |
| **Randomisation** | | | | | | | | | |
| **Sequence generation** | **8a** | yes | good | no | | yes | good | no | |
| | **8b** | yes | ok | yes | ok | yes | good | yes | ok |
| **Allocation** | **9** | yes | ok | yes | ok | yes | good | no | |
| **Implementation** | **10** | no | | no | | yes | ok | no | |
| **Blinding** | **11a** | NR | | NR | | NR | | NR | |
| | **11b** | NR | | NR | | NR | | NR | |
| **Statistical methods** | **12a** | yes | good | yes | good | yes | good | yes | ok |
| | **12b** | NR | | NR | | NR | | NR | |
| **Results** | | | | | | | | | |
| **Participant flow** | **13a** | yes | ok | no | | yes | good | no | |
| | **13b** | yes | ok | no | | yes | good | no | |
| **Recruitment** | **14a** | yes | good | yes | good | yes | good | yes | good |
| | **14b** | NR | | NR | | NR | | NR | |
| **Baseline data** | **15** | yes | ok | no | | no | | no | |
| **Numbers analysed** | **16** | yes | ok | yes | bad | yes | good | yes | bad |
| **Outcomes & estimation** | **17a** | yes | good | yes | good | yes | ok | yes | ok |
| | **17b** | yes | good | yes | good | NR | | NR | |
| **Ancillary analyses** | **18** | NR | | NR | | NR | | NR | |
| **Harms** | **19** | no | | no | | no | | no | |
| **Discussion** | | | | | | | | | |
| **Limitations** | **20** | yes | ok | yes | good | yes | good | yes | ok |
| **Generalisability** | **21** | yes | ok | yes | ok | yes | ok | yes | ok |
| **Interpretation** | **22** | yes | good | yes | good | yes | good | yes | good |
| **Other information** | | | | | | | | | |
| **Registration** | **23** | yes | | no | | no | | no | |
| **Protocol** | **24** | no | | no | | no | | no | |
| **Funding** | **25** | yes | | yes | | yes | | yes | |

**S3 Appendix. Quality assessment of included studies using the CONSORT 2010 checklist as a framework.** NR: Not Relevant. Continued.
