# Supplementary material for: Environmental methods for dengue vector control – A systematic review and meta-analysis
Source: PLoS Negl Trop Dis. 2019 Jul 11;13(7):e0007420. doi: 10.1371/journal.pntd.0007420 (PMC6650086; doi:10.1371/journal.pntd.0007420)
Supplement: S6 Appendix — (PDF) [file pntd.0007420.s006.pdf]

S6 Appendix. Formulas for difference-in-differences and difference-of-endlines calculation

The effect measures difference-in-difference (DID) and difference-of-endlines (DOE) were calculated for BI and PPI in each study using measures at baseline (b) and endline (e) separated by intervention (I) and control (C) group according to the following formulas.

$BI_{DID} = (BI_{ei} – BI_{bi}) – (BI_{ec} – BI_{bc})$

$PPI_{DID} = (PPI_{ei} – PPI_{bi}) – (PPI_{ec} – PPI_{bc})$

$BI_{DOE} = BI_{ei} – BI_{ec}$

$PPI_{DOE} = PPI_{ei} – PPI_{ec}$
