# Supplementary material for: Environmental methods for dengue vector control – A systematic review and meta-analysis
Source: PLoS Negl Trop Dis. 2019 Jul 11;13(7):e0007420. doi: 10.1371/journal.pntd.0007420 (PMC6650086; doi:10.1371/journal.pntd.0007420)
Supplement: S1 Table — (PDF) [file pntd.0007420.s007.pdf]

#	Author	Setting	Study design	Interventions	Control	Sample size	Follow-up	Outcome measures	Results	Study conclusions
**15**	**Overgaard 2016**	Colombia (Anapoima and La Mesa)	cRCT, 2×2 factorial	**Container covers:** fitting lids or nets on all water storage containers, containers that could not be fitted were treated with pyriproxyfen (once every two months)<br>**Waste management with direct garbage collection:** weekly solid waste clean-up and collection campaigns<br>**Others:** insecticide-treated curtains	Untreated control	9 intervention and control clusters of one school each	Duration: 3 months<br>Follow-ups after 3, 8, 15 and 21 months	HI, CI, BI, PPI<br>Density of adult female *Ae. aegypti* mosquitoes per hour<br>Number of pupil absence episodes	Dengue interventions significantly reduced the mean BI by almost 80%<br>Endline: CI and PPI were lower in intervention cluster (2.2 and 0.04) compared to control clusters (7.1 and 0.36)<br>Mean density of adult female *Ae. aegypti*: 1 - 2 per hour, no significant differences between intervention and control group<br>No significant differences between study groups for school absence	Efficacy of the dengue interventions is clearly shown, either alone or in combination with diarrheic interventions<br>Simultaneous interventions in schools and communities could improve outcomes of integrated approaches in the future
**16**	**Andersson 2015**	Nicaragua Managua Mexico (Guerrero)	cRCT, pragma-tic open label parallel group	**Waste management without direct garbage collection:** discussion of baseline evidence, community events to raise awareness, clean-up campaigns, information and teaching through brigadistas<br>**Others:** introduction of fish into water storage containers (Mexico only) and further activities	Routine control programme	75 intervention and control clusters of about 140 households each	Duration: 4 months<br>Follow-up in Nicaragua after 12 and 17 months and in Mexico after 12 and 15 months	HI, CI, BI, PPI<br>Paired saliva samples from children aged 3-9<br>Reported cases of dengue in the past year	Reductions in all entomological indices in intervention clusters<br>Relative risk reduction:<br>HI: 44.1 (95% CI: 13.6 to 74.7)<br>CI: 36.7 (95% CI: 24.5 to 44.8)<br>BI: 35.1 (95% CI: 16.7 to 55.5)<br>PPI: 51.7 (95% CI: 36.2 to 76.1)<br>Lower risk of dengue virus infection in children	Additional effectiveness of dengue control by evidence based community mobilisation<br>Future research on integrating vector control strategies within primary healthcare<br>Importance of community mobilisation and integrated management
**17**	**Basso 2015**	Uruguay (Salto)	cRCT	**Elimination:** elimination of small water containers, protection or removal of existing tanks, campaign for the physical or functional removal of containers in and around their homes, provision of container collection bags<br>(only 1 day for the collection/elimination)<br>**Container covers:** for large water tanks	Routine control programme	10 intervention and control clusters of about 100 households each	Duration: 2 months<br>Follow-up after 2 months	HI, CI, BI, PPI<br>Number of positive containers	Increase in vector densities in both arms due to seasonal variations (from spring to autumn) with lower indices in intervention clusters<br>BI increased in intervention clusters from 3.4 to 12.02 and in control clusters from 2.64 to 13.77<br>PPI increase in intervention clusters from 2.7 times and in control clusters 8.7 times	Evidence that the intervention can reduce dengue vector densities<br>Seasonal variations lead to fluctuations in temperature and resulted in higher vector densities at endline<br>Future tasks: implementation of the intervention in much larger geographic areas for a better quantification of the effectiveness

S1 Table. Evidence table. Summary of studies included in the systematic review and meta-analysis. The table only refers to dengue-related data. Unless otherwise stated, the follow-up was calculated starting with implementation of the intervention. #49e-h are not included in systematic review and meta-analysis.

#	Author	Setting	Study design	Interventions	Control	Sample size	Follow-up	Outcome measures	Results	Study conclusions
**18**	**Caprara 2015**	Brazil (Fortaleza)	cRCT	**Container covers:** for the elevated containers<br>**Waste management with direct garbage collection:** community workshops, community involvement in clean-up campaigns, in-house rubbish disposal without larviciding, mobilisation of schoolchildren and senior inhabitants and distribution of information, education and communication (IEC) materials in the community	Routine control programme	10 intervention and control clusters of about 100 households each	Intervention development: 4months<br>Follow-up after 16 months	HI, CI, BI, PPI	All entomological indices increased from baseline (dry season) to endline (rainy season) bit the increase was significantly higher in control clusters<br>For example, HI increased in intervention clusters from 1.3 to 2.05 and in control clusters from 0.84 to 3.17 (similar increases of all other indices)	Effectiveness of the ecohealth programme: significant reduction of the dengue vector population through targeted interventions in the most productive container types<br>Embedding environmental management and social participation can significantly reduce dengue vector densities<br>Seasonal variations resulted in higher vector densities at endline
**19**	**Mitchell-Foster 2015**	Ecuador (Machala)	cRCT	**Waste management without direct garbage collection:** integrated intervention strategy (IIS):<br>social mobilisation strategies, elementary school-based dengue education programme (DESE), and clean patio and safe container programme	Routine control programme	10 intervention and control clusters of about 100 households each	Duration: 12 months<br>Follow-up after 12 months	HI, BI, PPI	Significantly reduced overall PPI values were observed in the intervention clusters (from 0.52 to 0.08) following the IIS implementation period compared to control clusters (from 0.82 to 0.35)<br>Effect of the interventions was significantly stronger compared to the routine programme (OR 2.2, 95% CI 1.2 to 4.7)<br>DESE arm: reductions in HI (from 13.0 to 1.3) and BI (from 29.6 to 1.7) in intervention households	Integration of successful social mobilisation and empowerment strategies with biolarvicide-based government dengue prevention and control programmes is promising in reducing PPI and dengue transmission risk<br>Further implementation trials would be beneficial in terms of sustainability<br>Importance of integrating efforts, empowerment and intersectoral collaboration
**20**	**Quintero 2015**	Colombia, Girardot	cRCT	2 timely separated interventions:<br>1) **Others:** long-lasting insecticide-treated net windows and door curtains<br>2) **Container covers with insecticides**	Routine control programme	10 intervention and control clusters of at least 100 households each	First intervention: Duration: 2 months<br>Follow-up after 9 weeks<br>Second intervention (3 months after the first one): Duration: 6 months<br>Follow-up 4-6 weeks after the intervention	HI, CI, BI, PPI<br>Number of positive containers	First intervention: greater reduction in all indices in intervention clusters (only significance for BI)<br>BI decrease in intervention clusters by 57% and in control clusters by 38%<br>Second intervention:<br>Significant reductions in intervention clusters for PPI (71%) compared to control clusters (25%)	The intervention package reduced dengue vector densities<br>To achieve long-lasting behavioural changes, appropriate social mobilisation should be enhanced

S1 Table: Evidence table. Continued.

#	Author	Setting	Study design	Interventions	Control	Sample size	Follow-up	Outcome measures	Results	Study conclusions
**21**	**Abeyewickreme 2012**	Sri Lanka (Gampaha district)	cRCT	**Waste management with direct garbage collection:** building partnerships of local stakeholders, waste management at household level, the promotion of composting biodegradable household waste, raising awareness on the importance of solid waste management in dengue control and improving garbage collection with the assistance of local government authorities	Routine control programme	4 intervention and control clusters of about 200 households each	Duration: 12 months<br>Follow-up after 3, 9 and 15 months	HI, CI, BI, PPI	PPI lower in intervention clusters at any follow-up with a mean reduction of 98.2% after 15 months<br>No differences in HI and CI between the arms in any follow-up<br>The mean BI dropped from 11.75 and 9.75 at baseline to 3.13 and 6.25 after 15 months follow-up in intervention and control clusters, respectively	Community mobilisation was essential for implementation and sustainability<br>Importance of coordinating local authorities with increased household responsibility
**22**	**Arunachalam 2012**	India (Chennai city)	cRCT	**Container covers:** distribution of water container covers (in the context of a clean-up campaign<br>**Waste management with direct garbage collection:** community-based environmental management approach like provision of water container covers through community actors, clean-up campaigns, dissemination of dengue information through schoolchildren, distribution of information, education and communication materials in the community, mobilisation of existing women's self-help groups, management of waste disposal and recycling	Routine control programme	10 intervention and control clusters of about 100 households each	Duration: 10 months<br>Follow-up after 5 and 10 months	HI, CI, BI, PPI	Significant reductions in all entomological indices in intervention clusters after 10 months<br>PPI significantly decreased from 1.075 to 0.004 in the intervention group<br>HI, CI and BI reduced from 19.6 to 4.2, from 8.91 to 1.05 and from 30.8 to 4.3	Substantial reductions in dengue vector densities<br>Importance of community involvement
**23**	**Castro 2012**	Cuba (La Lisa Municipality in West Havana)	cRCT	**Waste management with direct garbage collection:** four components on top of routine control: organisation and management, entomological risk surveillance, capacity building, and community work for vector control	Routine control programme	16 intervention and control clusters (circumscriptions)	Duration: 30 months<br>Follow up: use of routine entomological surveillance data collected between Jan 2004 and Dec 2007	BI	At baseline, BI was comparable between the study groups<br>At the end: *A. aegypti* infestation levels increased but were significantly lower in intervention clusters than those in control clusters<br>Difference over the intervention period: BI was 53% lower in intervention clusters	The intervention was effective compared to routine control measures<br>Increased community involvement through empowerment

S1 Table: Evidence table. Continued.

#	Author	Setting	Study design	Interventions	Control	Sample size	Follow-up	Outcome measures	Results	Study conclusions
**24**	**Kittayapong 2012**	Thailand (Chachoeng-sao Province)	cRCT	**Container covers:** screen net covers (MosNetH) for water jars<br>**Waste management with direct garbage collection:** health education, mobilisation of dengue vector control activities, information of householders and provision of materials, cleaning solid waste in or around houses<br>**Elimination:** Source reduction and environmental management, such as getting rid of discarded or unused water holding containers<br>**Others:** *Mesocyclops aspericornis*, *Bti* sacs, mosquito traps and portable vacuum aspirators	No information about control group	10 intervention and control clusters with a total of 441 and 448 households	Duration: 6 months<br>Follow-up after 2, 4 and 6 months	HI, CI, BI, PPI<br>Number of positive containers	Significant reductions in PPI in the intervention group<br>PPI decreased in intervention clusters from 0.44 to 0.04 and in control clusters from 0.42 to 0.4<br>After 6 months, larval indices decreased in all clusters with no significant differences between the arms	Successful implementation of an eco-friendly vector control programme<br>Significant reductions in dengue vector densities<br>Success of community-based programmes depends on people awareness and understanding<br>Importance of long-lasting community participation with support from local authorities
**25**	**Rizzo 2012**	Guatemala (Poptun)	cRCT	2 timely separated interventions:<br>1) **Others:** coverage of windows and exterior doorways made of PermaNet 2.0 netting, factory treated with deltamethrin at 55 mg/m2, and of 200 L drums with similar treated material<br>2) **Container covers:** coverage of drums with insecticide-treated materials<br>**Elimination:** emptying buckets and elimination of disposable items<br>**Others:** treatment of productive containers with Temephos	Routine control programme	10 intervention and control clusters of about 100 households each	First intervention:<br>Follow up after 6 weeks<br>Second intervention (17 months after the first intervention):<br>Follow-up after 6 weeks	HI, CI, BI, PPI	First intervention: PPI increased with higher levels in control areas<br>Second intervention: borderline significant reduction of PPI and BI, significant reduction of HI and no significant difference of CI between intervention and control clusters<br>PPI: mean change in intervention clusters was -0.01 (95% CI: -0.26 to 0.24) and in control clusters was 0.52 (95% CI: -0.01 to 1.05)	Combination of methods can reduce vector densities significantly<br>Underestimation of productive containers in the wet season where the interventions were implemented, because the baseline survey was performed during dry season
**26**	**Tana 2012**	Indonesia (Yogyakarta)	cRCT	**Waste management without direct garbage collection:** community involvement and empowerment through meetings, forums, community leaders, neighbourhood assemblies, women associations, primary schools, production of communication materials and development of awareness campaigns in schools	Routine control programme	2 intervention and control clusters of about 100 households each	Duration: 12 months<br>Follow-up after 17 months	HI, CI, BI, PPI	BI declined from 6.9 to 0.5 in intervention clusters and increased from 2.0 to 3.5 in control clusters<br>Similar differences of -0.01 for PPI in intervention and control group	Community-centred multi-stakeholder approach has a higher chance for sustainability than a vertical “top-down” approach<br>Long-term effectiveness remains to be investigated

S1 Table: Evidence table. Continued.

#	Author	Setting	Study design	Interventions	Control	Sample size	Follow-up	Outcome measures	Results	Study conclusions
**27**	**Wai 2012**	Myanmar (Yangon city)	cRCT	**Container covers:** lid covers<br>**Waste management with direct garbage collection:** eco-friendly multi-stakeholder partner groups, ward-based volunteers, informed decision-making of householders, followed by integrated vector management approach, provision of waste-collection bags<br>**Others:** pyriproxyfen sand granules, *Bti*, cotton-net sweepers, dragon fly nymphs	Routine control programme	6 intervention and control clusters of about 100 households each	Duration: 6 months<br>Follow-up: 3 bi-monthly surveys	CI, BI, PPI	Lower entomological indices in control cluster at endline<br>PPI decreased by 32% in intervention clusters and by 54.5% in control clusters<br>Larval indices showed similar results	Effectiveness of the intervention was not shown: the study areas were hit by a cyclone and as a response, massive larviciding with Temephos was launched in control clusters<br>In terms of sustainability, community-controlled partnership-driven interventions are superior to vertical approaches
**28**	**Kusumawathie 2009**	Sri Lanka (Degaldoru-wa in Kandy district)	RCT	**Container covers:** application of plastic net covers, education on the correct use	Untreated control	46 intervention and control tanks	Duration: 6 months<br>Follow-up after 6 months	Number of positive containers	Significant reduction in the mean number of tanks positive for Aedes larvae in the intervention group (from 63 to 7) compared to the control group (from 33 to 31)<br>(t = -2.9616, p = 0.01; 95% CI 0.991 to 7.008)	Plastic net covers are effective, less costly and user friendly for the prevention of *Aedes* mosquitoes breeding in ground level cemented water tanks<br>A proper use of net covers is important
**29a**	**Tun-Lin 2009**	Mexico (Merida)	cRCT	**Waste management:** buckets and pot management	Blanket interven-tion	9 intervention and control clusters of about 100 households each	Duration: 5 months<br>Follow-up after 1 and 5 months	BI, PPI	Reduction from baseline in %:<br>BI intervention group: 47.6%, BI control group: 117.0%, PPI intervention group: -44.4%, PPI control group: 235.3%	Targeting only the most productive container types was as effective than targeting all water holding containers
**29b**	**Tun-Lin 2009**	Peru (Iquitos)	cRCT	**Elimination:** source reduction<br>**Others:** pyriproxyfen in productive containers	Blanket interven-tion	10 intervention and control clusters of about 50 households each	Duration: 5 months<br>Follow-up after 1 and 5 months	BI, PPI	Reduction from baseline in %:<br>BI intervention group: 4.1%, BI control group: 16.5%, PPI intervention group: 236.4%, PPI control group: -47.6%	Targeting only the most productive container types was as effective than targeting all water holding containers
**29c**	**Tun-Lin 2009**	Philippines (Quezon)	cRCT	**Waste management**<br>**Others:** tire splitting, drum and dish rack cleaning	Blanket interven-tion	8 intervention and 9 control cluster of 90-100 households each	Duration: 5 months<br>Follow-up after 1 and 5 months	BI, PPI	Reduction from baseline in %:<br>BI intervention group: -80.3%, BI control group: -75.8%, PPI intervention group: -73.2%, PPI control group: -73.1%	Targeting only the most productive container types was as effective than targeting all water holding containers
**29d**	**Tun-Lin 2009**	Venezuela (Trujillo)	cRCT	**Container covers with insecticides:** covering drums	Blanket interven-tion	9 intervention and control clusters of about 80 households each	Duration: 5 months<br>Follow-up after 1 and 5 months	BI, PPI	Reduction from baseline in %:<br>BI intervention group: 25.9%, BI control group: 30.3%, PPI intervention group: -6.6%, PPI control group: -13.0%	Targeting only the most productive container types was as effective than targeting all water holding containers

S1 Table: Evidence table. Continued.

#	Author	Setting	Study design	Interventions	Control	Sample size	Follow-up	Outcome measures	Results	Study conclusions
29e	***Tun-Lin 2009***	*Kenya (Malindi)*	*cRCT*	*Temephos in productive containers*	*Blanket intervention*	*10 intervention and control clusters of 60 households each*	*Duration: 5 months*<br>*Follow-up after 1 and 5 months*	*BI, PPI*	*No specific results available*	*Targeting only the most productive container types was as effective than targeting all water holding containers*
29f	***Tun-Lin 2009***	*Thailand (Chachoeng-sao, Chiang Mai, Salsabury)*	*cRCT*	*Bti and pyriproxyfen in productive containers*	*Blanket intervention*	*9 intervention and control clusters of about 100 households each*	*Duration: 5 months*<br>*Follow-up after 1 and 5 months*	*BI, PPI*	*Reduction from baseline in %:*<br>*BI intervention group: -51.8%, BI control group: -51.0%, PPI intervention group: -14.8%, PPI control group: -48.6%*	*Targeting only the most productive container types was as effective than targeting all water holding containers*
29g	***Tun-Lin 2009***	*Myanmar (Yangon)*	*cRCT*	*Sweep method, Dragon-fly nymphs, fish*	*Blanket intervention*	*10 intervention and control clusters of 90-100 households each*	*Duration: 5 months*<br>*Follow-up after 1 and 5 months*	*BI, PPI*	*Reduction from baseline in %:*<br>*BI intervention group: -82.2%, BI control group: -81.8%, PPI intervention group: -76.3%, PPI control group: -78.4%*	*Targeting only the most productive container types was as effective than targeting all water holding containers*
29h	***Tun-Lin 2009***	*Vietnam (Binh Thuan province)*	*cRCT*	*Mesocyclops in productive containers*	*Blanket intervention*	*9 intervention and control clusters of 70 households each*	*Duration: 5 months*<br>*Follow-up after 1 and 5 months*	*BI, PPI*	*No specific results available*	*Targeting only the most productive container types was as effective than targeting all water holding containers*
**30**	**Vanlerberghe 2009**	Cuba (Guantana-mo)	cRCT	**Waste management without direct garbage collection:** community based environmental management approach: discussion of the intervention with local stakeholders and formation of a local steering committee, creation of formal task forces at grassroots level to secure community involvement in environmental management, strengthening of intersectoral coordination<br>**Elimination:** source reduction through periodic house inspections	Routine control programme	16 intervention and control clusters (circumscriptions) of about 500 households each	Duration: 10 months<br>Follow-up after 10 months	HI, BI, PPI	Entomological indices were comparable at baseline and significantly lower in intervention clusters at endline<br>*Aedes* foci were lower in intervention clusters than those in control clusters (50% lower for HI and BI, 73% lower for PPI)	Effectiveness of a community based environmental management programme in reducing dengue vector indices<br>Future proceedings: extension of the intervention to the whole province
**31**	**Toledo 2007**	Cuba (Santiago de Cuba)	cRCT, quasi-experi-mental design	**Container covers**<br>**Waste management with direct garbage collection:** promotion of specific behavioural change and reduction of environmental risks through social communication strategies and intersectoral local government activities	Routine control programme	20 intervention and control clusters (family medicine practices and the neighbourhoods they cover)	Duration: 24 months<br>Follow-up after 12 and 24 months	HI, CI<br>Number of positive containers	Significant reductions in the median CIs and HIs and in the number of positive containers similar in intervention and control group<br>For example, HI decreased from 1.23 to 0.35 in intervention clusters and from 2.08 to 0.52 in control clusters	Similar effectiveness of dengue vector control strategy in intervention clusters compared with routine control activities<br>Importance of local task forces representing both, the interests of householders and vector control workers

S1 Table: Evidence table. Continued.

#	Author	Setting	Study design	Interventions	Control	Sample size	Follow-up	Outcome measures	Results	Study conclusions
**32a**	**Kroeger 2006**	Mexico (Veracruz, Agua Dulce)	cRCT	**Container covers with insecticides:** curtains treated with lambdacyhalothrin and water treatment with pyriproxyfen chips (an insect growth regulator)	Untreated control	9 intervention and control cluster with a mean of 61 houses per sector	Duration: 14 months<br>Follow-up after 2-4 weeks, 4 months and 12 months	HI, CI, BI, PPI<br>IgM serology	At endline, entomological indices were significantly lower with no significant differences between intervention and control group<br>For example, the BI decreased from 60 to 7 in intervention clusters and from 113 to 12 in control clusters	Use of insecticide treated covers can reduce the number of dengue vectors and thereby reduce disease transmission.<br>Temporal trends in vector densities in intervention areas might have caused bias<br>Larger trials implementing insecticide-treated materials were started in Asia and Latin America
**32b**	**Kroeger 2006**	Venezuela (Trujillo)	cRCT	**Container covers with insecticides:** curtains treated with longlasting deltamethrin (PermaNet) plus water jar covers of the same material	Untreated control	9 intervention and control cluster with a mean of 62 houses per sector	Duration: 14 months<br>Follow-up after 2-4 weeks, 4 months and 12 months	HI, CI, BI, PPI<br>IgM serology	At endline, entomological indices were significantly lower with no significant differences between intervention and control group<br>For example, the BI decreased from 38 to 11 in intervention clusters and from 34 to 17 in control clusters	Use of insecticide treated covers can reduce the number of dengue vectors and thereby reduce disease transmission.<br>Temporal trends in vector densities in intervention areas might have caused bias<br>Larger trials implementing insecticide-treated materials were started in Asia and Latin America
**33**	**Leontsini 1993**	Honduras (El Progreso)	cRCT	**Waste management without direct garbage collection:** information meetings about dengue and its prevention for community leaders and interested members, formation of committees for planning activities and household visits, community clean-up campaigns and sewage system maintenance work	Untreated control	4 intervention and control clusters with a total of 1800 households	Duration: 6 months<br>Follow-up after 3 and 7 months	HI, BI	Relative reductions of 35% and 43% in BI for intervention and control clusters<br>Similar results for HI	Measurable effect of the community intervention on larval production sites<br>Larger scale interventions were planned in order to increase sustainability

S1 Table: Evidence table. Continued.
