# Supplementary figures and images for: Environmental methods for dengue vector control – A systematic review and meta-analysis
Source: PLoS Negl Trop Dis. 2019 Jul 11;13(7):e0007420. doi: 10.1371/journal.pntd.0007420 (PMC6650086; doi:10.1371/journal.pntd.0007420)

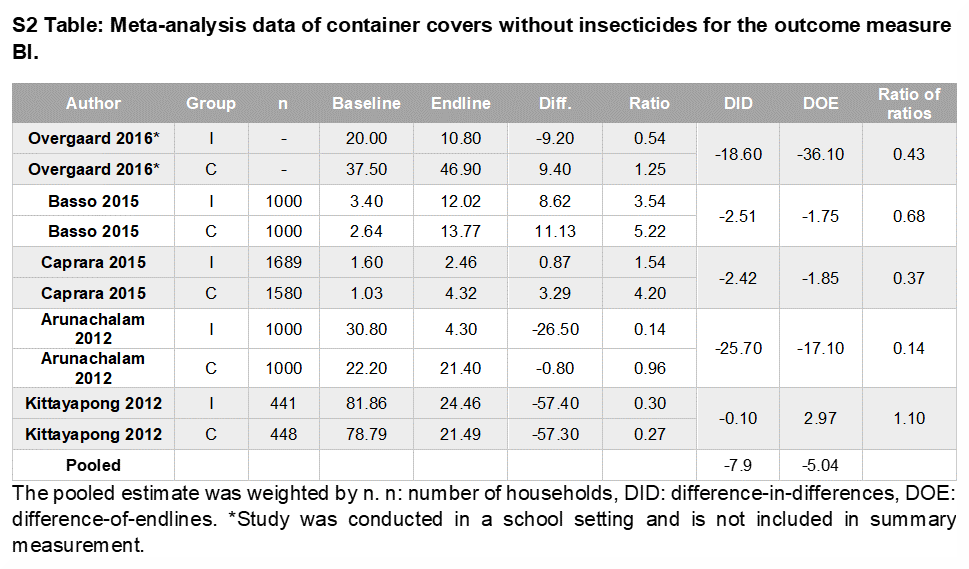

Supplement: S2 Table — (TIF) [file pntd.0007420.s008.tif]

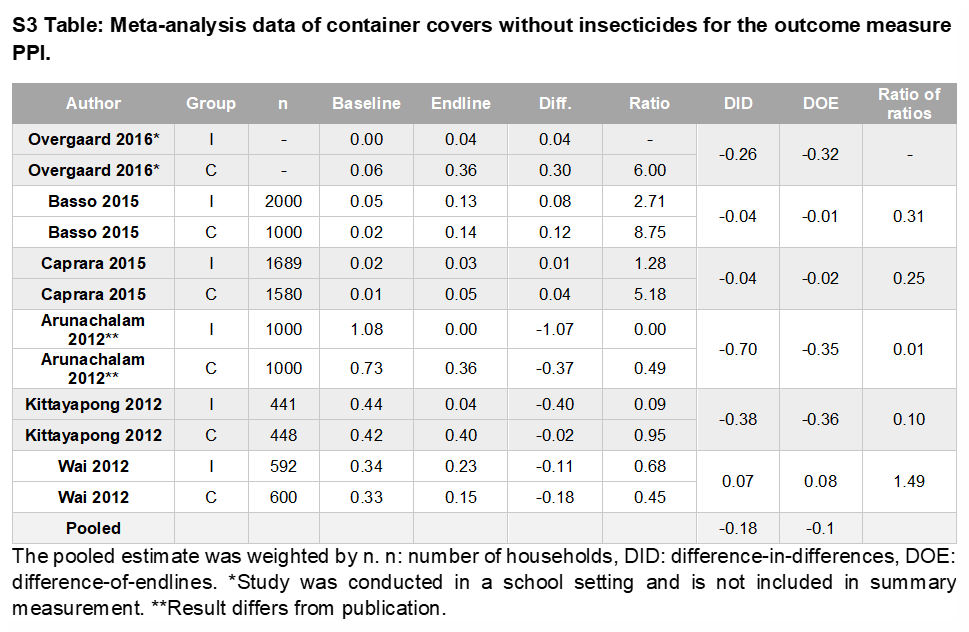

Supplement: S3 Table — (TIF) [file pntd.0007420.s009.tif]

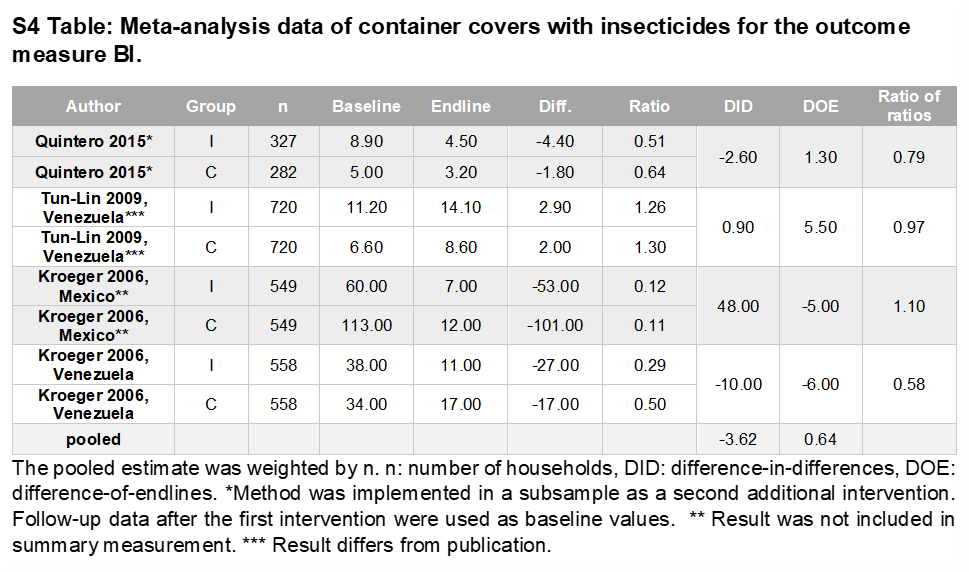

Supplement: S4 Table — (TIF) [file pntd.0007420.s010.tif]

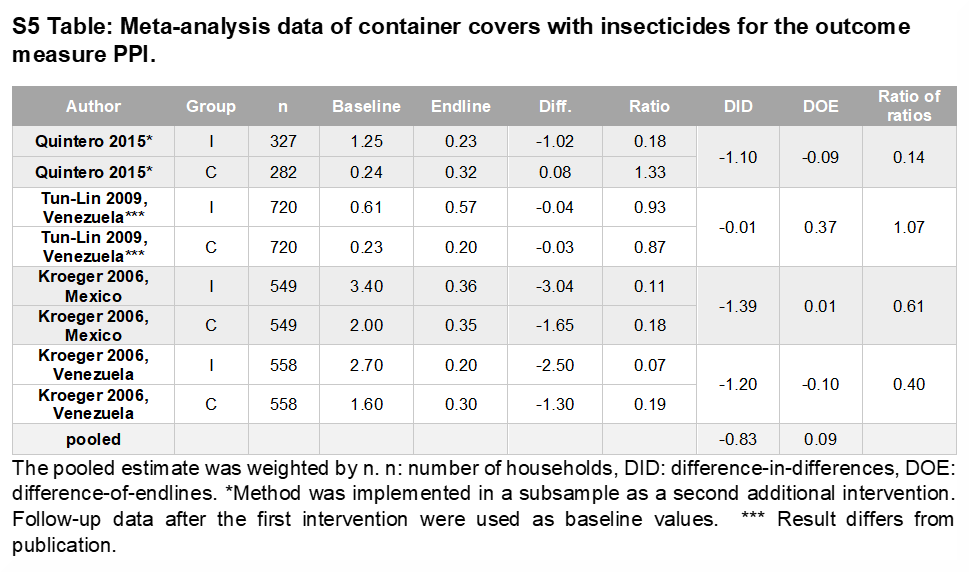

Supplement: S5 Table — (TIF) [file pntd.0007420.s011.tif]

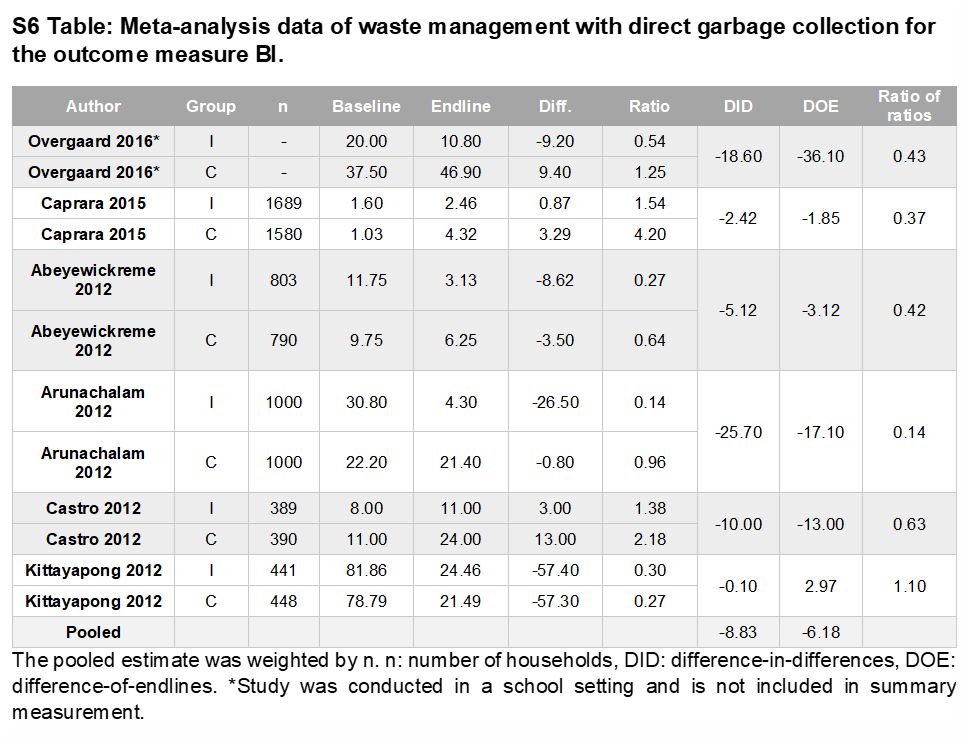

Supplement: S6 Table — (TIF) [file pntd.0007420.s012.tif]

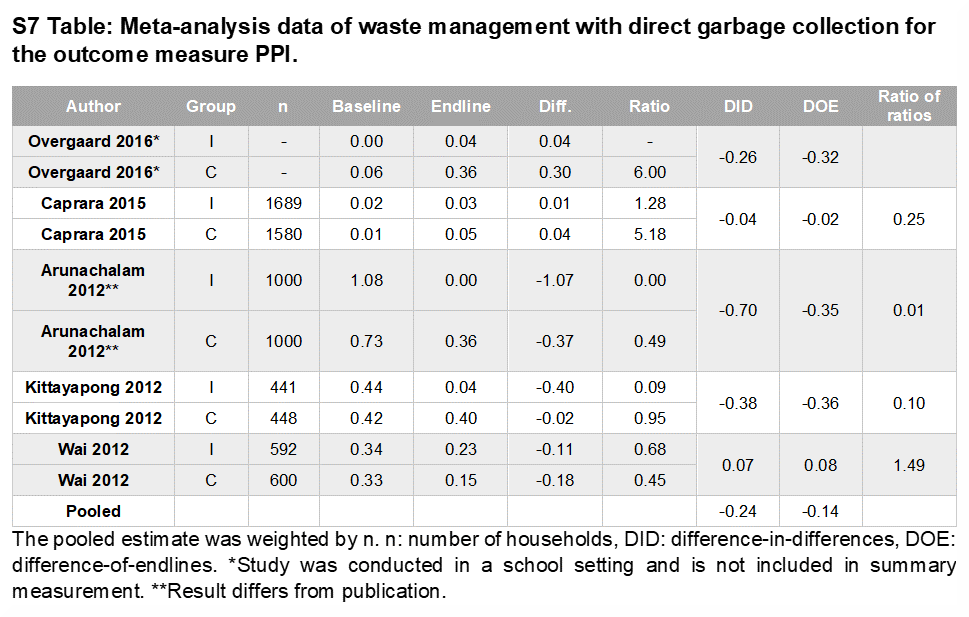

Supplement: S7 Table — (TIF) [file pntd.0007420.s013.tif]

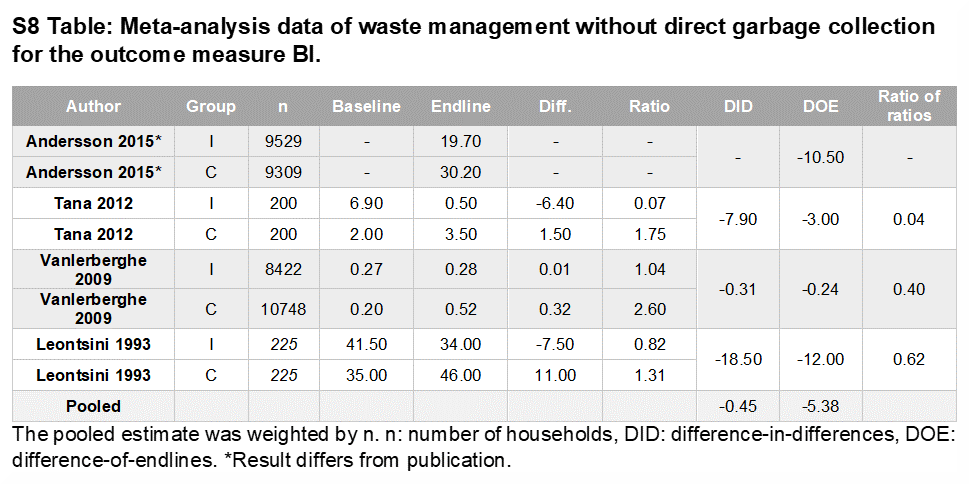

Supplement: S8 Table — (TIF) [file pntd.0007420.s014.tif]

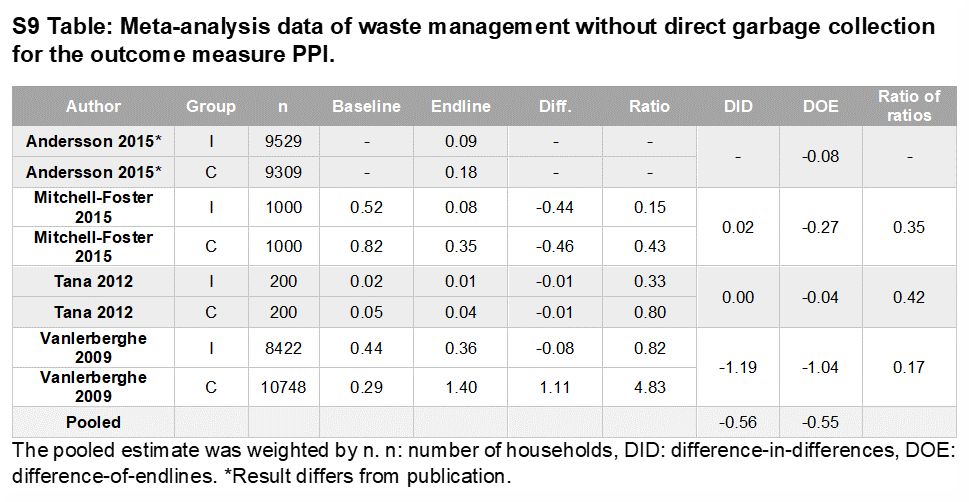

Supplement: S9 Table — (TIF) [file pntd.0007420.s015.tif]

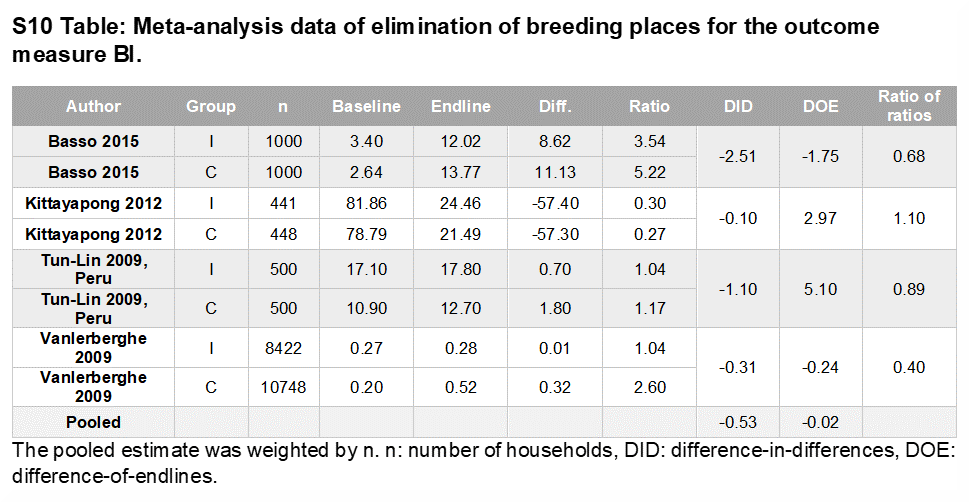

Supplement: S10 Table — (TIF) [file pntd.0007420.s016.tif]

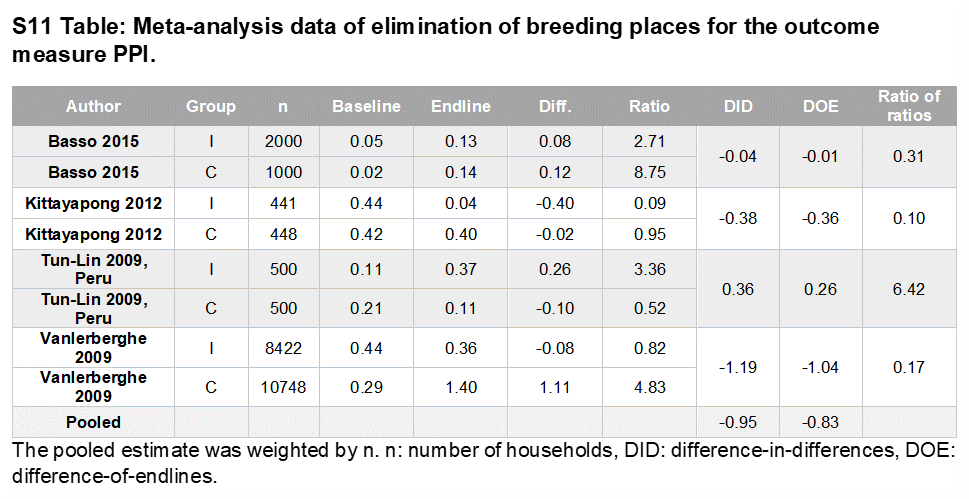

Supplement: S11 Table — (TIF) [file pntd.0007420.s017.tif]

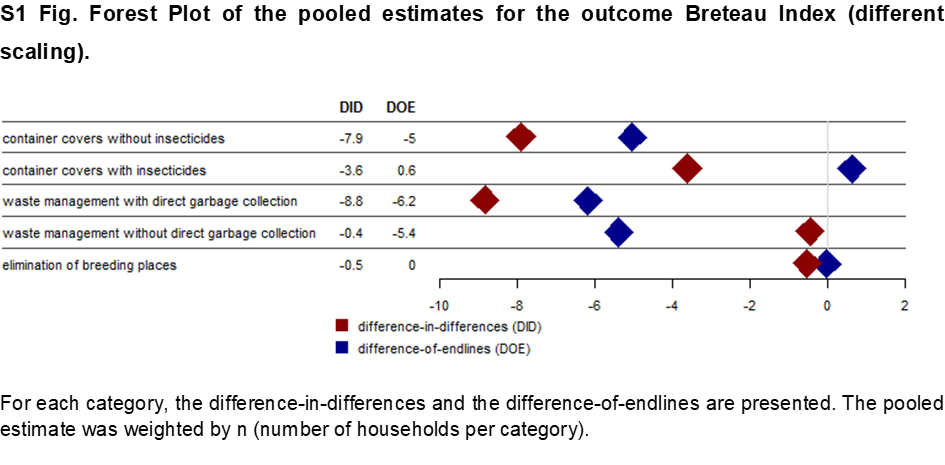

Supplement: S1 Fig — (TIF) [file pntd.0007420.s018.tif]

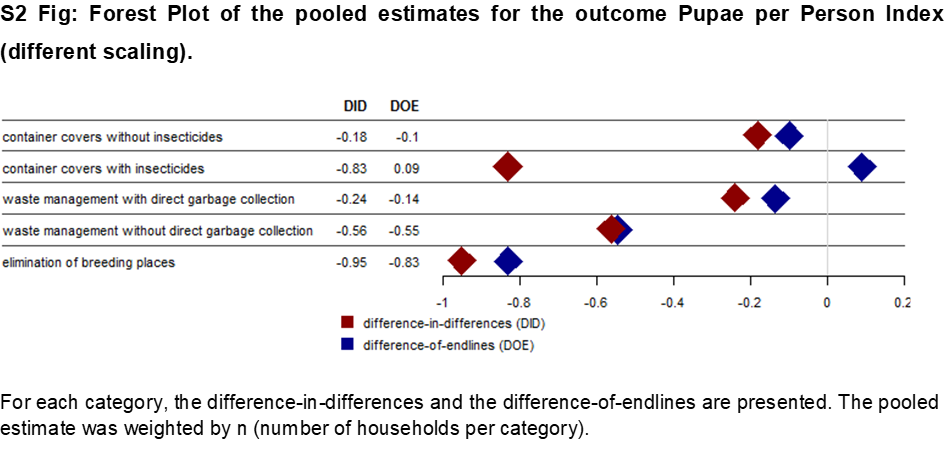

Supplement: S2 Fig — (TIF) [file pntd.0007420.s019.tif]
